# HCV Core Residues Critical for Infectivity Are Also Involved in Core-NS5A Complex Formation

**DOI:** 10.1371/journal.pone.0088866

**Published:** 2014-02-12

**Authors:** Katarzyna Gawlik, James Baugh, Udayan Chatterji, Precious J. Lim, Michael D. Bobardt, Philippe A. Gallay

**Affiliations:** Department of Immunology and Microbial Science, The Scripps Research Institute, La Jolla, California, United States of America; University of Washington, United States of America

## Abstract

Hepatitis C virus (HCV) infection is a major cause of liver disease. The molecular machinery of HCV assembly and particle release remains obscure. A better understanding of the assembly events might reveal new potential antiviral strategies. It was suggested that the nonstructural protein 5A (NS5A), an attractive recent drug target, participates in the production of infectious particles as a result of its interaction with the HCV core protein. However, prior to the present study, the NS5A-binding site in the viral core remained unknown. We found that the D1 domain of core contains the NS5A-binding site with the strongest interacting capacity in the basic P38-K74 cluster. We also demonstrated that the N-terminal basic residues of core at positions 50, 51, 59 and 62 were required for NS5A binding. Analysis of all substitution combinations of R50A, K51A, R59A, and R62A, in the context of the HCVcc system, showed that single, double, triple, and quadruple mutants were fully competent for viral RNA replication, but deficient in secretion of viral particles. Furthermore, we found that the extracellular and intracellular infectivity of all the mutants was abolished, suggesting a defect in the formation of infectious particles. Importantly, we showed that the interaction between the single and quadruple core mutants and NS5A was impaired in cells expressing full-length HCV genome. Interestingly, mutations of the four basic residues of core did not alter the association of core or NS5A with lipid droplets. This study showed for the first time that basic residues in the D1 domain of core that are critical for the formation of infectious extracellular and intracellular particles also play a role in core-NS5A interactions.

## Introduction

Hepatitis C virus (HCV) is a member of the *Hepacivirus* genus within the *Flaviviridae* family, a group of small, enveloped, single-stranded RNA viruses [1]. HCV is a blood-born virus with the propensity to establish a chronic liver infection that can result in steatosis, liver fibrosis, cirrhosis and hepatocellular carcinoma [2]. Available treatment options are limited by both efficacy and tolerability even after the addition of newly approved protease inhibitors, boceprevir and telaprevir, to the standard of care consisting of ribavirin and pegylated alpha interferon [3]. Approximately 200 million people worldwide are currently infected with HCV and the annual rate of HCV-related hepatocellular carcinoma is projected to triple by 2030 [4]. Therefore, the development of more effective, less toxic, and ultimately interferon-free therapeutic approaches, is of paramount importance. This goal has become more and more attainable with a better understanding of the HCV life cycle [5].

HCV particles contain a positive polarity RNA genome with 5′ and 3′ untranslated regions (UTR) and a long open reading frame encoding a polyprotein precursor of about 3,000 amino acids. Translation of the polyprotein is initiated by ribosome binding to an internal ribosome entry site (IRES), which spans most of the 5′-UTR and the first 24–40 nucleotides of the core coding region [1], [6], [7]. This results in the production of a single precursor polyprotein, which is processed by cellular and viral proteases into 10 structural and nonstructural (NS) proteins (core, E1, E2, p7, NS2, NS3, NS4A, NS4B, NS5A and NS5B). Core protein, which forms the nucleocapsid, and the envelope glycoproteins (E1 and E2) make up the structural components of the virion. Nonstructural proteins from NS3 to NS5B are thought to assemble into a membranous-web-associated HCV RNA replication complex that catalyzes the amplification of the viral genome. Whereas RNA replication is independent of the structural proteins, the assembly and egress of infectious viral particles require p7, NS2, NS3, and NS5A, in addition to the structural components [8]. The development of the infectious HCV cell culture system (HCVcc) based on the genotype 2a strain called JFH1 and its derivatives allowed analysis of the essential contribution of nonstructural proteins and host cell factors to virion morphogenesis [9]–[13].

**Figure 1:**
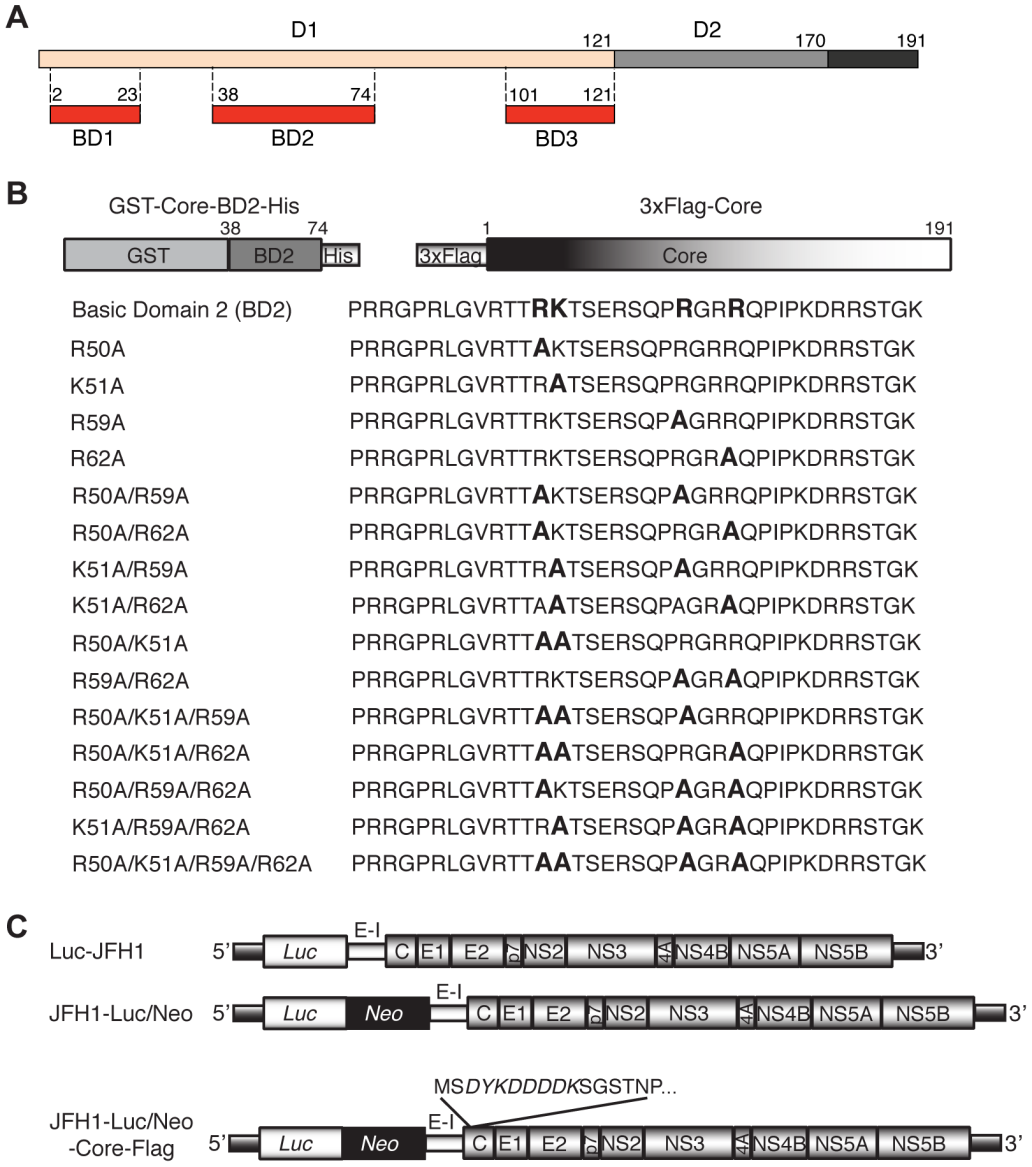
Schematic representation of the core protein and the constructs used in the study. A) The precursor core of 191 amino acids is processed by a signal peptide peptidase, giving a mature protein of around 170 amino acids that is composed of two domains, D1 and D2. Based on the charge distribution, the D1 domain can be subdivided into three basic clusters: BD1 (basic domain 1), BD2 (basic domain 2) and BD3 (basic domain 3). B) Two recombinant proteins were used to determine the requirement of basic residues R50, K51, R59 and R62 in core for NS5A binding. GST-Core-BD2-His was used as bait in pulldown assays, while full-length 3xFlag-Core was used in co-immunoprecipitations in SGR-JFH1 cells. A sequence of wild-type BD2 as well as a series of single, double, triple and quadruple alanine substitutions in its context is listed below. C) A scheme of luciferase reporter full-length JFH1 genomes (genotype 2a) used in the study. Previously described Luc-JFH1 [12], [61] was used to generate JFH1-Luc/Neo construct by insertion of a neomycin-resistant gene (black box) after the luciferase cassette (white box), but before EMCV-IRES (E-I) and all structural and nonstructural HCV proteins (shadow boxes). The JFH1-Luc/Neo-Core-Flag construct was created by an insertion of a Flag tag (DYKDDDDK) with a short linker (SGS) between the amino acids S2 and T3 of core. Single, double, triple and quadruple alanine substitutions of core residues R50, K51, R59 and R62 listed above (Figure 1B) were introduced into the JFH1-Luc/Neo-Core-Flag. Additionally, the quadruple core mutant was created in the context of wild-type JFH1-Luc/Neo.

Although its major function is to encapsidate the HCV genome, core is a multifunctional protein reported to interact with a variety of cellular proteins and to influence numerous host cell functions such as gene transcription, lipid metabolism, apoptosis and cell signaling [14], [15]. The precursor core of 191 amino acids is processed by a signal peptide peptidase, giving a mature protein of 177 residues or so, which is targeted to lipid droplets (LDs) [16]–[18]. A visualization study of core trafficking during assembly in live virus producing cells identified core as polarized caps on immotile LDs and as small motile puncta along microtubules [19]. The three-dimensional structure of core is unknown. Circular dichroism analyses demonstrated that the mature core protein is a dimeric, alpha-helical protein that can be divided into two domains, D1 and D2 [20]. A study showed that the nucleocapsid-like particles of HCV most likely contain a dimer of core protein that is stabilized by a disulfide bond [21]. The D1 domain of core is rich in basic residues and is located at the N-terminal two-thirds of the core, whereas the D2 domain encompasses the C-terminus and is more hydrophobic. Based on the charge distribution of amino acids [22], the D1 domain of core can be subdivided into three basic clusters (Figure 1A): the basic domain 1 (BD1; 2–23 aa), the basic domain 2 (BD2; 38–74 aa), and the basic domain 3 (BD3; 101–121 aa). The D1 domain is mainly involved in viral RNA binding [23], [24] and oligomerization necessary for particles formation [25]–[27]. The D2 domain is responsible for core association with LDs and with endoplasmic reticulum (ER) membranes [28].

A current model of HCV assembly couples the viral particle formation with the cellular lipid metabolism [11], [29]–[31]. The HCV assembly process appears to be spatially associated with LDs, the storage sites for neutral lipids in cells. Studies using the HCVcc system demonstrated that LDs are required for the formation of infectious viral particles and indicated a key role of core and NS5A in this process [32], [33]. Mutations of core and NS5A, which abolish the targeting of both proteins to LDs, impair virus production [32]. It has been proposed that core recruits NS proteins, HCV RNA and the replication complex to LD-associated membranes. It has also been proposed that core recruits NS5A to LDs through direct interactions involving three serine residues at positions 2428, 2430, 2433 of NS5A (amino acid position in the context of the HCV polyprotein) [34]. Importantly, several studies identified domains I and II of NS5A as sites for RNA binding [35]–[38]. Thus, NS5A plays a dual role in RNA replication and assembly processes as a potential switch between these two critical viral steps [39]–[42]. The recent discovery of a potent anti-HCV compound BMS-790052, an NS5A inhibitor, turned NS5A into a highly attractive target for drug development, even though its mode of action is still unknown [43], [44]. In addition to NS5A, other NS proteins such as NS2, NS3, NS4A and NS4B were shown to be critical for HCV assembly [45]–[51]. Furthermore, host factors were also shown to be implicated in the HCV assembly process as the tail interacting protein of 47 kDa (TIP47) and diacylglycerol acetyltransferase-1 (DGAT1) [52]–[54]. Apolipoprotein E (ApoE) was detected in infectious HCV virions [55], [56] and showed to be essential for assembly, most probably via a specific interaction with NS5A [57], [58].

Core and NS5A are both able to bind viral RNA and are both considered to be key players in HCV assembly. The direct interaction between them through the C-terminal serine cluster of NS5A was proposed to be involved in infectious virus particles formation [34]. However, the regions and residues of core, responsible for this interaction, remain unknown. In this study, we examined whether core-NS5A interactions truly occur in the infectious HCVcc system and investigated which regions and residues of core are responsible for NS5A contacts. We found that indeed core and NS5A form complexes in a relevant HCV infectious system. We showed that core-NS5A interactions are direct and that the D1 domain of core contains the NS5A-binding site. Importantly, the BD2 of core (the basic P38-K74 cluster) contains the major NS5A-binding site. Basic residues R50, K51, R59, and R62 located in BD2 were shown to be crucial for NS5A binding. Importantly, mutating the examined basic residues abolishes both infectivity and release of HCV particles. Altogether, the results presented in this study, further emphasize the critical role of core-NS5A interactions in the HCV life cycle.

## Materials and Methods

### Cells and cell culture

Human hepatoma Huh7 and Huh7.5.1 cells [13] were maintained in Dulbecco's modified Eagle's medium (DMEM) containing 10% fetal bovine serum (FBS) and supplemented with nonessential amino acids (NEAA), 2 mM L-glutamine, 10 mM HEPES, 100 units/ml penicillin, and 100 mg/ml streptomycin (Invitrogen). Huh7 cells carrying a subgenomic JFH1 replicon (SGR-JFH1) were maintained in the same medium as mentioned above and supplemented with 300 μg/ml G418 (BioPioneer) [59]. Huh7.5.1 and SGR-JFH1 cells were kindly provided by Francis V. Chisari. Huh7 and SGR-JFH1 cells were used for co-immunoprecipitation experiments. Huh7.5.1 cells were used for HCV RNA transfection and infection assays. To generate stable cell lines, Huh7.5.1 cells were transfected with full-length genomic JFH1-Luc/Neo constructs and 48 h post-transfection 250 μg/ml of G418 was added to medium. Culture medium supplemented with G418 at the same concentration was replaced twice a week. After 2 weeks, emerging colonies were pooled and kept under continuous G418 pressure for 1–2 weeks in order to establish full-length JFH1 stable cell lines. The maintenance of the mutations in all of the stable cell lines was verified by RT-PCR and sequencing. Total RNA was purified from cells using RNasy Plus Mini Kit (Qiagen). Synthesis of the first strand cDNA was performed using SuperScript III First-Strand Synthesis System for PT-PCR (Invitrogen) and gene specific primer 5′-CACCGGGCTGCCGCACAGCCATGTTTGGCGAGACTGGCA-3′. Obtained cDNA was used as a template to amplify full-length core by PCR with a pair of oligonucleotides 5′-AGTCAAATGGCTCTCCTCAAGCGT and 5′-AGCAGAGACCGGAACGGTGATGCA-3′ spanning EMCV-IRES and core nucleotide sequence (positions 3475 and 4213 of JFH1-Luc/Neo replicon, respectively). The authenticity of the constructs was confirmed by sequencing of PCR products.

### Plasmids construction

The plasmid pJFH1 encoding full-length JFH1 genome of genotype 2a (GenBank accession number AB047639) described previously [12], [13] was used as template to clone full-length NS5A and NS5A constructs as well as all core constructs by PCR mutagenesis with primers listed in Table 1. For bacterial expression, the NS5A PCR products were cloned into SacII and HindIII sites in pET-UbCHis vector [60], whereas for mammalian expression, into HindIII and EcoRI sites in the pcDNA3 vector. Core PCR products were cloned into BamHI and EcoRI sites in the pGEX-2T vector for bacterial expression system as well as into the same restriction sites in pcDNA3. Cloning of the 3xFlag-Core construct for mammalian expression was performed in two steps. The first step created core N-terminally fused with a single Flag tag (DYKDDDDK) that was used as template for the second step that created core with 3xFlag tag (DYKDHDGDYKDHDJDYKDDDDK). The GST-BD2-His in pGEX-2T and 3xFlag-Core in pcDNA3 were further used as templates to create a series of mutants with single, double, triple and quadruple alanine substitutions of core amino acids R50A, K51A, R59A, and R62A by site-directed mutagenesis (QuickChange II XL Site-Directed Mutagenesis Kit; Agilent Technologies).

**Table 1:** Oligonucleotide sequences.

Name	Sequence
NS5A-His	5′-GCGGGTACCCCGCGGTGGATCCGGATCCTGGCTCCGC GAC-3′
	5′-GCGGGTACCAAGCTTCTTGTCATCGTCGTCCTTGTAGTC GCAGCACACGGTGGTATCGTC-3′
Domain I NS5A-Flag	5′-GCGGGTACCCCGCGGTGGATCCGGATCCTGGCTCCGC GAC-3′
	5′-GCGGGTACCAAGCTTCTTGTCATCGTCGTCCTTGTAGTC AGTCTCCGCCGTGATGTGGGG-3′
Domain II NS5A-Flag	5′-GCGGGTACCCCGCGGTGGATATGACGTGGACATGGTC GAT-3′
	5′-GCGGGTACCAAGCTTCTTGTCATCGTCGTCCTTGTAGTC ACAACCAGCAACGGTGGGCGG-3′
Domain III NS5A-Flag	5′-GCGGGTACCCCGCGGTGGAAGGAGACGCCGGACAGTG GGT-3′
	5′-GCGGGTACCAAGCTTCTTGTCATCGTCGTCCTTGTAGTC GCAGCACACGGTGGTATCGTC-3′
Amphipathic Helix NS5A-Flag	5′- GCGGGTACCCCGCGGTGGATCCGGATCCTGGCTCCGC GAC-3′
	5′-GCGGGTACCAAGCTTCTTGTCATCGTCGTCCTTGTAGTC GGGCAGCTTGGGGAACAATTT-3′
Subdomain IA NS5A-Flag	5′-GCGGGTACCCCGCGGTGGAGGCCTCCCCTTCATCTCT TGT-3′
	5′-GCGGGTACCAAGCTTCTTGTCATCGTCGTCCTTGTAGTC CGGCGCGCACTGGCCCTCCGT-3′
Subdomain IB NS5A-Flag	5′-GCGGGTACCCCGCGGTGGAAAACCCCCCACGAACTAC AAG-3′
	5′-GCGGGTACCAAGCTTCTTGTCATCGTCGTCCTTGTAGTC AGTCTCCGCCGTGATGTGGGG-3′
NS5A (pcDNA3)	5′-CACAAAGCTTCCACCATGTCCGGATCCTGGCTCCGC GAC-3′
	5′-CACAGAATTCTTAGCAGCACACGGTGGTATCGTC-3′
GST-Core1-40-His	5′-CACAGGATCCATGAGCACAAATCCTAAACCT-3′
	5′-CACAGAATTCTTAATGGTGATGATGATGGTGACCTCCCC CAGAACCTCCCCCCCTGCGCGGCAACAAGTATAC-3′
GST-Core1-80-His	5′-CACAGGATCCATGAGCACAAATCCTAAACCT-3′
	5′-CACAGAATTCTTAATGGTGATGATGATGGTGACCTCCCC CAGAACCTCCCCCACCTGGTTTTCCCCAGGCCTT-3′
GST-Core1-100-His	5′-CACAGGATCCATGAGCACAAATCCTAAACCT-3′
	5′-CACAGAATTCTTAATGGTGATGATGATGGTGACCTCCCC CAGAACCTCCCCCGGGGGACAGGAGCCATCCTGC-3′
GST-Core1-170-His	5′-CACAGGATCCATGAGCACAAATCCTAAACCT-3′
	5′-CACAGAATTCTTAATGGTGATGATGATGGTGACCTCCCC CAGAACCTCCCCCGGGTAGGTTCCCTGTTGCATA-3′
GST-Core24-170-His	5′-CACAGGATCCTTCCCGGGCGGCGGCCAGATC-3′
	5′-CACAGAATTCTTAATGGTGATGATGATGGTGACCTCCCC CAGAACCTCCCCCGGGTAGGTTCCCTGTTGCATA-3′
GST-Core75-170-His	5′-CACAGGATCCGCCTGGGGAAAACCAGGTCGC-3′
	5′-CACAGAATTCTTAATGGTGATGATGATGGTGACCTCCCC CAGAACCTCCCCCGGGTAGGTTCCCTGTTGCATA-3′
GST-Core122-170-His	5′-CACAGGATCCGTCATCGACACCCTAACGTGT-3′
	5′-CACAGAATTCTTAATGGTGATGATGATGGTGACCTCCCC CAGAACCTCCCCCGGGTAGGTTCCCTGTTGCATA-3′
GST-Core2-23-His	5′-CACAGGATCCAGCACAAATCCTAAACCTCAA-3′
	5′-CACAGAATTCTTAATGGTGATGATGATGGTGACCTCCCC CAGAACCTCCCCCCTTAACGTCTTCTGGGCGACG-3′
GST-Core38-74-His	5′-CACAGGATCCCCGCGCAGGGGCCCCAGGTTG-3′
	5′-CACAGAATTCTTAATGGTGATGATGATGGTGACCTCCCC CAGAACCTCCCCCCTTGCCAGTGGAGCGCCGATC-3′
GST-Core101-121-His	5′-CACAGGATCCCGAGGCTCTCGCCCCTCCTGG-3′
	5′-CACAGAATTCTTAATGGTGATGATGATGGTGACCTCCCC CAGAACCTCCCCCTTTACCCACGTTGCGCGACCT-3′
Flag-Core (pcDNA3)	5′-CACAGGATCCATGGACTACAAGGACGACGATGACAAG GGGGGAGGTATGAGCACAAATCCTAAACCT-3′
	5′-CACAGAATTCTTAAGCAGAGACCGGAACGGTGAT-3′
3xFlag-Core (pcDNA3)	5′-CACAGGATCCACCATGGACTACAAAGACCATGACGGTGAT TATAAAGATCATGACATCGATTACAAGGATGACGATGACAAG-3′
	5′-CACAGAATTCTTAAGCAGAGACCGGAACGGTGAT-3′
JFH1-Luc/Neo-Core-Flag (pFK)	5′-AAACACGATGATACCATGAGCGACTACAAGGACGACGATG ACAAGTCTGGATCTACAAATCCTAAACCTCAAAGA-3′
	5′-TCTTTGAGGTTTAGGATTTGTAGATCCAGACTTGTCATCGT CGTCCTTGTAGTCGCTCATGGTATCATCGTGTTT-3′
Core R50A	5′-GGTGTGCGCACGACAGCGAAAACTTCGGAGCGG-3′
	5′-CCGCTCCGAAGTTTTCGCTGTCGTGCGCACACC-3′
Core K51A	5′-GTGCGCACGACAAGGGCAACTTCGGAGCGGTCC-3′
	5′-GGACCGCTCCGAAGTTGCCCTTGTCGTGCGCAC-3′
Core R59A	5′-GAGCGGTCCCAGCCAGCTGGGAGACGCCAGCCC-3′
	5′-GGGCTGGCGTCTCCCAGCTGGCTGGGACCGCTC-3′
Core R62A	5′-CAGCCACGTGGGAGAGCCCAGCCCATCCCCAAA-3′
	5′-TTTGGGGATGGGCTGGGCTCTCCCACGTGGCTG-3′
Core R50A/K59A	5′-TTGGGTGTGCGCACGACAGCGAAAACTTCGGAGCGGT CCCAGCCAGCTGGGAGACGCCAGCCCATCCCCAAAGAT-3′
	5′-ATCTTTGGGGATGGGCTGGCGTCTCCCAGCTGGCTGGG ACCGCTCCGAAGTTTTCGCTGTCGTGCGCACACCCAA-3′
Core R50A/R62A	5′-TTGGGTGTGCGCACGACAGCGAAAACTTCGGAGCGGT CCCAGCCACGTGGGAGAGCCCAGCCCATCCCCAAAGAT-3′
	5′-ATCTTTGGGGATGGGCTGGGCTCTCCCACGTGGCTGGG ACCGCTCCGAAGTTTTCGCTGTCGTGCGCACACCCAA-3′
Core K51A/R59A	5′-TTGGGTGTGCGCACGACAAGGGCAACTTCGGAGCGGT CCCAGCCAGCTGGGAGACGCCAGCCCATCCCCAAAGAT-3′
	5′-ATCTTTGGGGATGGGCTGGCGTCTCCCAGCTGGCTGGG ACCGCTCCGAAGTTGCCCTTGTCGTGCGCACACCCAA-3′
Core K51A/R62A	5′-TTGGGTGTGCGCACGACAAGGGCAACTTCGGAGCGGT CCCAGCCACGTGGGAGAGCCCAGCCCATCCCCAAAGAT-3′
	5′-ATCTTTGGGGATGGGCTGGGCTCTCCCACGTGGCTGGG ACCGCTCCGAAGTTGCCCTTGTCGTGCGCACACCCAA-3′
Core R50A/K51A	5′-TTGGGTGTGCGCACGACAGCGGCAACTTCGGAGCGGT CCCAGCCACGTGGGAGACGCCAGCCCATCCCCAAAGAT-3′
	5′-ATCTTTGGGGATGGGCTGGCGTCTCCCACGTGGCTGGG ACCGCTCCGAAGTTGCCGCTGTCGTGCGCACACCCAA-3′
Core R59A/R62A	5′-TTGGGTGTGCGCACGACAAGGAAAACTTCGGAGCGGT CCCAGCCAGCTGGGAGAGCCCAGCCCATCCCCAAAGAT-3′
	5′-ATCTTTGGGGATGGGCTGGGCTCTCCCAGCTGGCTGGG ACCGCTCCGAAGTTTTCCTTGTCGTGCGCACACCCAA-3′
Core R50A/K51A/R59A	5′-TTGGGTGTGCGCACGACAGCGGCAACTTCGGAGCGGT CCCAGCCAGCTGGGAGACGCCAGCCCATCCCCAAAGAT-3′
	5′-ATCTTTGGGGATGGGCTGGCGTCTCCCAGCTGGCTGGG ACCGCTCCGAAGTTGCCGCTGTCGTGCGCACACCCAA-3′
Core R50A/K51A/R62A	5′-TTGGGTGTGCGCACGACAGCGGCAACTTCGGAGCGGT CCCAGCCACGTGGGAGAGCCCAGCCCATCCCCAAAGAT-3′
	5′-ATCTTTGGGGATGGGCTGGGCTCTCCCACGTGGCTGGG ACCGCTCCGAAGTTGCCGCTGTCGTGCGCACACCCAA-3′
Core R50A/R59A/R62A	5′-ATCTTTGGGGATGGGCTGGGCTCTCCCAGCTGGCTGGG ACCGCTCCGAAGTTTTCGCTGTCGTGCGCACACCCAA-3′
	5′-ATCTTTGGGGATGGGCTGGGCTCTCCCAGCTGGCTGGG ACCGCTCCGAAGTTTTCGCTGTCGTGCGCACACCCAA-3′
Core K51A/R59A/R62A	5′-TTGGGTGTGCGCACGACAAGGGCAACTTCGGAGCGGT CCCAGCCAGCTGGGAGAGCCCAGCCCATCCCCAAAGAT-3′
	5′-ATCTTTGGGGATGGGCTGGGCTCTCCCAGCTGGCTGGG ACCGCTCCGAAGTTGCCCTTGTCGTGCGCACACCCAA-3′
Core R50A/K51A/R59A/R62A	5′-TTGGGTGTGCGCACGACAGCGGCAACTTCGGAGCGGT CCCAGCCAGCTGGGAGAGCCCAGCCCATCCCCAAAGAT-3′
	5′-ATCTTTGGGGATGGGCTGGGCTCTCCCAGCTGGCTGGG ACCGCTCCGAAGTTGCCGCTGTCGTGCGCACACCCAA-3′
GND	5′-ACAATGCTGGTATGCGGCAATGACCTAGTAGTCATCTCA-3′
	5′-TGAGATGACTACTAGGTCATTGCCGCATACCAGCATTGT-3′

Mutation sites are in bold.

The plasmid pFK-Luc-JFH1 was obtained from Thomas Pietschmann and described previously [12], [61]. The XbaI site in the firefly luciferase gene and the NotI site in the EMCV IRES were utilized to clone the Luciferase/Ubiquitin-NPT II (the neomycin phosphotransferase II gene) fusion cassette out of pFK389ILuc-NS (wild-type replicon from genotype 1b) [62], [63] and placed into the pFK-Luc-JFH1 plasmid, creating the full-length JFH1-Luc/Neo construct. An insertion of a single Flag tag (DYKDDDDK) with a short linker (SGS) between the amino acids S2 and T3 of core into the JFH1-Luc/Neo and an introduction of double, triple and quadruple alanine substitutions R50A, K51A, R59A, and R62A into the core gene of JFH1-Luc/Neo-Core-Flag and JFH1-Luc/Neo were constructed by site-directed mutagenesis (QuickChange II XL Site-Directed Mutagenesis Kit; Agilent Technologies). Sequences of oligonucleotides used for mutagenesis are listed in Table 1. The authenticity of the constructs was confirmed by DNA sequencing.

### Antibodies and reagents

The following antibodies were used in the study: anti-NS5A monoclonal antibody (9E10, a kind gift from Charles M. Rice); anti-Core antigen monoclonal antibody (C7-50, Thermo Scientific); 6xHis monoclonal antibody (Clontech); monoclonal anti-Flag M2 antibody (Sigma); GST•Tag monoclonal antibody (Novagen); β-Actin, rabbit monoclonal antibody (LI-COR Biosciences); anti-TIP47, rabbit polyclonal antibody (M6PRBP1, Proteintech); anti-Calnexin, rabbit IgG fraction of antiserum (Sigma); human anti-E1 MAb 1C4 (Innogenetics); anti-E2 antibody D3.7 (DAO5, a kind gift from Arvind Patel); goat anti-ApoE polyclonal antibody (Millipore); IRDye 800CW conjugated goat anti-mouse IgG, IRDye 680LT goat anti-rabbit IgG, IRDye 680RD donkey anti-goat IgG, IRDye 680RD goat anti-human IgG (all IRDye secondary antibodies were from LI-COR Biosciences); ECL anti-mouse IgG, horseradish peroxidase linked whole antibody from sheep (GE Healthcare). All restriction enzymes were obtained from New England Biolabs. AccuPrime *Pfx* DNA polymerase was from Invitrogen whereas T4 DNA ligase was from Roche Applied Science. All other reagents were purchased from Sigma, Fisher, or VWR unless indicated otherwise.

### Flag co-immunoprecipitation

For co-immunoprecipitation experiments, Huh7 and SGR-JFH1 cells were seeded (5x10^5^) onto 10 cm plates and cultured overnight. Plasmid DNA was transfected into cells using GeneJuice Transfection Reagent (Novagen) according to the manufacturer's protocol and cells were grown for 72 h. Alternatively, stable cell lines expressing full-length JFH1-Luc/Neo-Core-Flag and JFH1-Luc/Neo-Core-Flag-R50A/K51A/R59A/R62A were grown to 90% confluency (∼2×10^6^ per 10 cm plate). Cells were washed twice with ice-cold DPBS with Ca^2+^ and Mg^2+^ and lysed in 0.5 ml of lysis buffer (50 mM Tris-HCl pH 7.5; 150 mM NaCl, 1% Triton X-100; 10% glycerol; 5 mM MgCl_2_; 1 mM EDTA; 1 mM PMSF; complete mini EDTA-free protease inhibitor mixture). The lysate was centrifuged at 20,000× *g* for 40 min at 4°C. The supernatant fraction was incubated for 4 h with 40 µl of 50% anti-Flag M2 agarose bead slurry (Sigma). Beads were collected and washed three times with 0.5 ml of lysis buffer to remove the impurities. Beads were then incubated for 1 h with 50 µl of 0.15 mg/ml 3xFLAG peptide to elute Flag-tagged proteins. Samples were centrifuged and the supernatant fraction analyzed by Western blotting using anti-NS5A and anti-Core antibodies.

### Expression and purification of recombinant proteins

Recombinant full-length GST and GST-CypA were produced and purified as described previously [37], [64]. Expression of NS5A-His and NS5A-His-Flag constructs were performed in the BL21(DE3)pCG1 strain of *E. coli* (generous gift from Craig Cameron) in 400 ml of Circlegrow (MP Biomedicals) supplemented with 100 mg/ml kanamycin and 20 µg/ml chloramphenicol at 37°C. Bacteria were grown to an *A*_600_ of 0.8–1.0 before isopropyl β-d-thiogalactopyranoside (IPTG) was added to a final concentration of 1 mM. Bacteria were grown for an additional 3 h at 30°C, harvested and purified as described previously [60]. Expression of GST-Core-His constructs was performed in *E. coli* BL21 (DE3) in 400 ml of Circlegrow supplemented with 100 mg/ml ampicillin at 37°C. Bacteria were grown to an *A*_600_ of 0.8–1.0 before IPTG was added to a final concentration of 0.5 mM. Bacteria were grown for an additional 3 h at 30°C and pelleted by centrifugation in a Fiberlite F10–6x500y rotor at 6000 rpm for 20 min at 4°C. Pellets were resuspended in 25 ml of lysis buffer (50 mM Tris-HCl, pH 8.0, 100 mM NaCl, 10% glycerol, 1 mM DTT, complete mini EDTA-free protease inhibitor mixture (Roche Applied Science)). Lysozyme (5 mg/ml), 1x Bug Buster (Novagen), DNase I (40 mg/ml), and RNase A (40 mg/ml) were added for complete lysis. Lysates were centrifuged in a Fiberlite F21–8x50y rotor at 16,000× *g* for 30 min at 4°C. Supernatants were loaded to a His Bind resin (Novagen) charged with Ni^2+^, incubated for 1 h in the cold room with gentle agitation and washed with 15 resin volumes of wash buffer I (20 mM Tris-HCl, pH 8.0, 0.5 M NaCl, 0.5% Igepal CA-630, 10% glycerol, 1 mM DTT, 5 mM imidazole, complete mini EDTA-free protease inhibitor mixture) and then with 10 resin volumes of wash buffer II (the same as wash buffer I, but with 60 mM imidazole). Purified His-tagged proteins were eluted with 1.0 ml elution buffer (the same as wash buffer I, but with 1 M imidazole) and dialyzed against dialysis buffer (50 mM Tris-HCl, pH 7.5, 100 mM NaCl, 5 mM MgCl_2_, 10% glycerol, 0.5% Igepal CA-630, 1 mM DTT). Purified proteins were aliquoted and stored at −80°C.

### GST pulldown assay

Glutathione-Sepharose 4B beads (GE Healthcare Biosciences) were incubated for 2 h in binding buffer (50 mM Tris-HCl, pH 7.5, 100 mM NaCl, 5 mM MgCl_2_, 10% glycerol, 0.5% Igepal CA-630, 1 mM DTT) with 5 mg/ml BSA at 4°C and washed twice in binding buffer. Meanwhile, 4 µg of GST, GST-CypA, or GST-Core-His proteins was mixed with 10 µg of full-length NS5A-His or NS5A-His proteins (domain I, domain II, domain III or amphipathic helix, subdomain IA, and subdomain IB) in a total volume of 100 µl of binding buffer and incubated for 3 h at 4°C on wheel. Glutathione beads (20 µl) were added to the recombinant protein mixture and incubated for 1 h at 4°C, then washed three times with 400 µl of binding buffer. Beads were pelleted for 1 min at 1000× *g* in a microcentrifuge and bound material eluted with 30 µl of SDS-PAGE sample loading buffer, heated for 5 min, and frozen at −20°C. Bound material was analyzed by Western blotting using anti-GST, anti-NS5A, anti-Flag and anti-His antibodies. Additionally, recombinant GST and NS5A proteins were treated with 250 mg and 500 mg of RNase (Roche Applied Science), 1 U and 10 U of DNase (Roche Applied Science) or 2.5 U and 25 U of benzonase nuclease (Novagen), for 1 h at room temperature. Recombinant GST and NS5A proteins were also treated with 1, 5 or 10 µM of BMS-790052 and 0.5, 1, 2 or 4 µM of CsA for 30 min at 4°C. Treated proteins were then mixed and pulldowns performed as described above.

### Western blot analysis

Bound material from GST pulldowns was directly eluted with SDS-PAGE loading sample buffer (125 mM Tris-HCl, pH 6.8; 2% SDS; 5% β-mercaptoethanol; 0.001% bromophenol blue, 10% glycerol). Eluted material from Flag co-immunoprecipitations as well as sucrose fractions from density ultracentrifugation experiments were mixed with SDS-PAGE loading sample buffer giving the final concentration mentioned above. After SDS-PAGE electrophoresis in NuPAGE 4–12% Bis-Tris gel and protein transfer to Immobilon-FL PVDF membrane (Millipore), Western blot analysis was performed according to the instructions from LI-COR Biosciences with some modifications. Briefly, Immobilon-FL PVDF membrane was incubated with 10 ml Odyssey blocking buffer overnight at 4°C with gentle agitation. After the blocking step, the membrane was incubated for 2 h at room temperature with primary antibodies diluted in Odyssey blocking buffer supplemented with 0.2% Tween 20. The membrane was then washed three times for 10 min each with PBS plus 0.1% Tween 20 and incubated with respective secondary IRDye antibodies diluted in Odyssey blocking buffer supplemented with 0.2% Tween 20 for 1 h at room temperature protected from light. The membrane was washed three times for 10 min each with PBS plus 0.1% Tween 20, and once with only PBS, dried, visualized, and analyzed on the Odyssey IR imaging system.

### *In vitro* transcription and transfection of HCV RNA

Plasmids encoding JFH1 reporter viruses were purified by phenol-chloroform extraction (Fisher) and dissolved in RNase-free water. *In vitro* transcription reaction was performed using a MEGAscript kit (Ambion) according to the manufacturer's instructions. After 4 h of incubation at 37°C, transcription was terminated by RNA extraction with acidic phenol and chlorophorm (Fisher), precipitated with LiCl and dissolved in RNase-free water. The concentration was determined by absorbance at 260 nm. *In vitro* transcribed genomic JFH1 RNA was delivered to cells by electroporation as described previously [13]. Briefly, single-cell suspensions of Huh7.5.1 cells were prepared by trypsinization of monolayers and subsequent resuspension with complete DMEM. Cells were washed with DPBS twice, counted, and resuspended at 10^7^ cells per ml in ice-cold DPBS and kept on ice. 10 µg of viral RNA was mixed with 400 µl of cell suspension by pipetting, electroporated, immediately transferred to 30 ml of complete DMEM and seeded in 10 cm (10 ml) or 6-well (3 ml) plates. Electroporation conditions were 270 V, 950 µF and 100 ohms with a Gene Pulser Xcell system (Bio-Rad) and a cuvette with a gap width of 0.4 cm (Bio-Rad). To measure luciferase activity, cells were washed once with DPBS, lysed directly on the 6-well plate with 0.3 ml of ice-cold Cell Culture Lysis Reagent (Promega) and frozen. After centrifugation at 14,000 rpm for 5 min at 4°C, 20 µl of cell lysate was used to determine the luciferase activity by Luciferase Assay System (Promega) on a luminometer (Centro LB960; Berthold) for 10 s. All luciferase assays were performed at least in triplicates. For stable cell lines expressing full-length genomic JFH1, cells were seeded in 6-well plates in duplicates at a concentration of 10^5^ cells per well, and luciferase activity measured after 72 h.

### Quantification of HCV core protein

Intracellular and extracellular HCV core levels were quantified by an enzyme immunoassay (Ortho HCV antigen ELISA kit; Ortho Clinical Diagnostics distributed by Waco Chemicals) according to the instructions of the manufacturer. For extracellular core measurement, cells stably expressing full-length genomic JFH1 were seeded in 6-well plates in triplicates at the concentration of 10^5^ cells per well, and grown for 72 h in 3 ml of complete DMEM. Collected supernatants were centrifuged at 2000× *g* for 10 min at 4°C and frozen. Then, supernatants were either directly used for ELISA or diluted 1∶10 with complete DMEM prior to measurement. For intracellular core quantification, cells from 6-well plates were lysed after 72 h with 0.3 ml of ice-cold Cell Culture Lysis Reagent (Promega) and frozen. Lysates were cleared at 20,000× *g* for 10 min at 4°C and tested for core content by ELISA at a dilution of 1∶1000 or 1∶10000 in DPBS.

### Extra- and intracellular infectivity assay

For extracellular infectivity, fresh and clarified cell culture supernatants of 90% confluent stable cell lines expressing full-length genomic JFH1 grown for 72 h in complete DMEM without G418 were used to determine viral titers by a focus forming assay on Huh7.5.1 cells as described previously [65]. Cell supernatants were serially diluted 10-fold in complete DMEM, and 100 µl was used to infect 6×10^3^ Huh7.5.1 cells in 96-well plates. Infected cells were fixed at 72 h post-inoculation and immunostained with a mouse monoclonal anti-Core antibody (C7-50; diluted 1∶200), followed by an anti-mouse IgG, horseradish peroxidase linked whole antibody from sheep (diluted 1∶500) and diaminobenzidine substrate (DAB; Sigma). Viral titers were expressed as the number of focus-forming units (FFU) per milliliter of supernatant. For intracellular infectivity, cell lysates of stable cell lines expressing full-length genomic JFH1 grown for 72 h in complete DMEM without G418 were prepared as described previously [66]. Approximately 6×10^6^ cells (three 10 cm plates) were washed once with DPBS, harvested by trypsinization, resuspended in 0.6 ml of complete DMEM and subject to four rounds of freeze-thaw cycles in dry ice and in a 37°C water bath, respectively. Cell debris were removed by centrifugation at 14,000 rpm for 5 min and supernatants were tested for intracellular infectivity as described above.

### Lipid droplets isolation

LDs were isolated as described with some modifications [32], [67]. Cells at a confluency of 90% (five 10 cm plates) were harvested by trypsinization, washed with DPBS and counted. Cells (10^7^) were lysed in 0.6 ml of hypotonic buffer (50 mM Hepes, pH 7.4; 1 mM EDTA and 2 mM MgCl_2_ supplemented with protease inhibitors) with 70 strokes in a tight-fitting Dounce homogenizer. After centrifugation at 14,000 rpm for 5 min at 4°C, post-nuclear fractions were mixed with equal volumes (0.5 ml) of 1.05 M sucrose (35%) in isotonic buffer (50 mM Hepes, 100 mM KCl, 2 mM MgCl_2_, protease inhibitors) and overlaid onto 3 ml of 60% sucrose in SW55Ti (Beckman) centrifuge tubes. Additionally, 1 ml of isotonic buffer was loaded onto the sucrose mixtures and centrifuged at 100,000× *g* for 2 h at 4°C in a SW55 Ti rotor (Beckman). After centrifugation, the LD fraction of 0.3 ml from the very top of the gradient solution was collected. Proteins of this fraction were precipitated with trichloroacetic acid, washed once with acetone, resuspended in SDS-PAGE sample loading buffer with urea (125 mM Tris-HCl, pH 6.8; 4% SDS; 5% β-mercaptoethanol; 0.001% bromophenol blue, 10% glycerol; 8 M urea) and analyzed by Western blotting.

### Separation of intracellular HCVcc particles using sucrose gradient ultracentrifugation

Intracellular HCVcc particles were partially purified as described previously [66]. Cells at a confluency of 90% (five 10 cm plates) were harvested by trypsinization, washed with DPBS and counted. Cells (10^7^) were lysed in 1.2 ml of TNE buffer (10 mM Tris-HCl, pH 8.0; 150 mM NaCl; 2 mM EDTA supplemented with protease inhibitors) with 70 strokes in a tight-fitting Dounce homogenizer. After centrifugation at 14,000 rpm for 5 min, post-nuclear fractions (1 ml) were deposited onto the top of a continuous 10 to 60% sucrose gradient prepared in the same TNE buffer. Equilibrium was reached by ultracentrifugation at 29,000 rpm for 16 h at 4°C in a SW41Ti rotor (Beckman). Samples of 1 ml were collected from the top of the gradient. Each fraction was analyzed for infectivity (100 µl) and protein content by Western blotting (10 µl). Fraction densities were determined by measuring the sucrose content in each fraction of a control gradient with a refractometer. Infectivity was assessed by a focus-forming assay on Huh7.5.1 cells as described above. For RT-PCR analysis, RNA was extracted by acidic phenol-chloroform (Fisher) extraction of the sucrose fractions (200 µl) followed by LiCl precipitation. Presence of viral RNA in each fraction was tested using 300 ng of RNA with two pairs of oligonucleotides using the SuperScript III One Step RT-PCR kit (Invitrogen), as per manufacturer's instructions. First pair of oligonucleotides 5′-AGTCAAATGGCTCTCCTCAAGCGT and 5′-AGCAGAGACCGGAACGGTGATGCA-3′ was spanning EMCV-IRES and core nucleotide sequence (positions 3475 and 4213 of JFH1-Luc/Neo replicon, respectively). Second pair of nucleotides 5′-TTCCGGGATGAGGTCTCGTTC-3′ and 5′-CTCTGTCTGAGCCACACCGCC-3′ was spanning NS5A nucleotide sequence (positions 10092 and 10377 of JFH1-Luc/Neo replicon, respectively).

## Results

### The NS5A-binding site is located in the D1 domain of core

**Figure 2:**
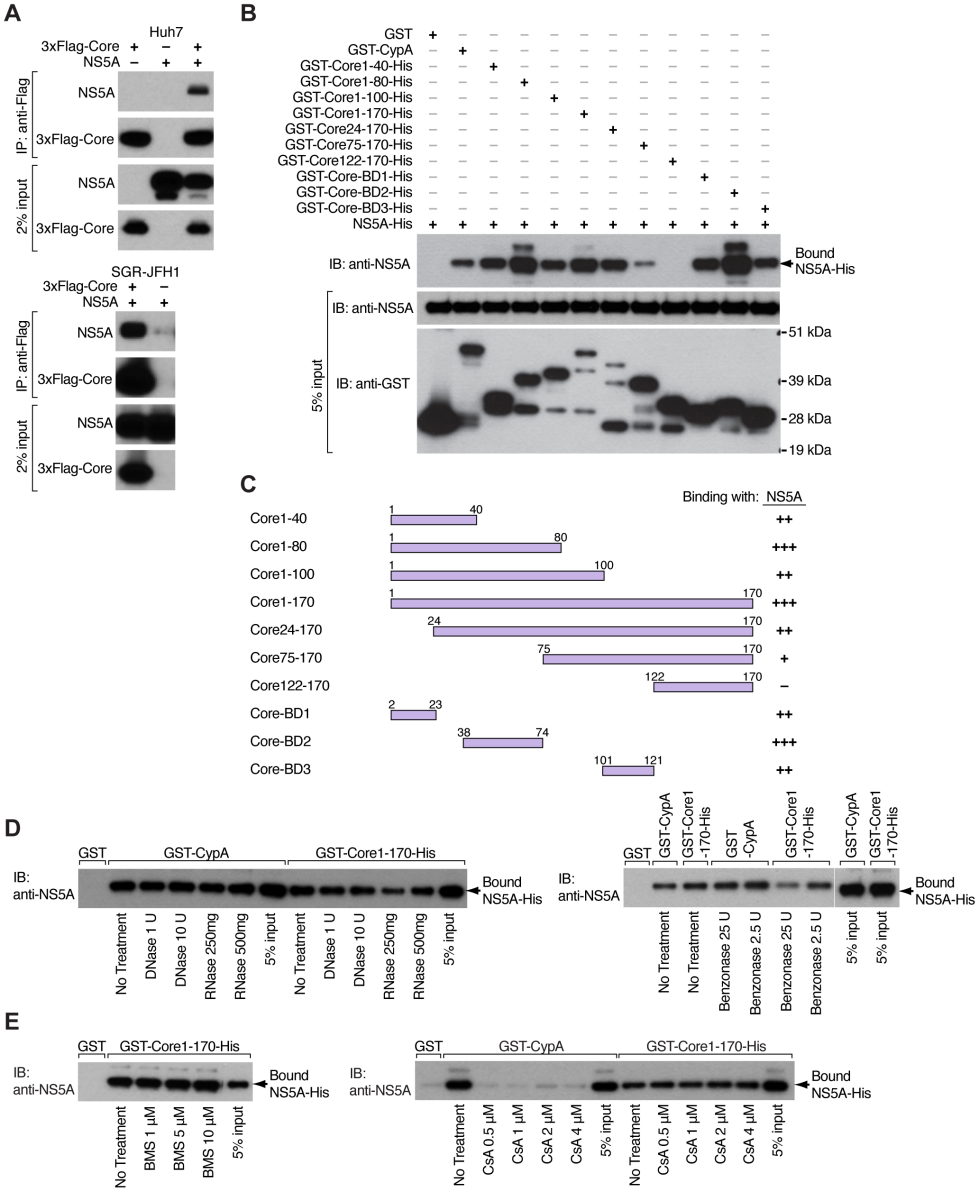
NS5A-binding site is located in the D1 domain of core. A) Flag co-immunoprecipitations in Huh7 cells transfected with expression vectors for 3xFlag-Core and NS5A (top panel) or in SGR-JFH1 (Huh7 subgenomic JFH1 replicon) cells transfected with an expression vector for 3xFlag-Core (bottom panel). After immunoprecipitation with anti-Flag antibodies, bound material was eluted with 3xFlag peptide and analyzed by Western blotting with anti-NS5A and anti-Core antibodies. Input of whole-cell lysate (2%) used for each co-immunoprecipitation was probed with anti-NS5A and anti-Core antibodies. B) Mapping of core regions required for NS5A binding. GST (negative control), GST-CypA (positive control) or truncated forms of GST-Core-His were used as bait to pulldown full-length NS5A-His. Captured proteins were analyzed by Western blotting using anti-NS5A antibodies. Input (5%) used for each GST pulldown was probed with anti-NS5A and anti-GST antibodies. C) Schematic representation of core regions required for binding to NS5A. The strongest interaction was expressed as (+++), less strong as (++), weak as (+) and no interaction as (−). The amount of NS5A bound by truncated forms of GST-Core-His was estimated relative to the amount of NS5A bound by GST-Core1-170-His, that showed the highest binding capacity in spite of the lowest level of input. D) Recombinant proteins (GST, GST-CypA, GST-Core1-170-His and NS5A-His) were treated with RNase and DNase (left panel) or benzonase nuclease (right panel) to remove contaminating nucleic acids before pulldown assays. GST-CypA/NS5A-His mixtures were used as controls because this interaction has been shown to be direct. Captured proteins were analyzed by Western blotting using anti-NS5A antibodies. E) Recombinant GST, GST-CypA, GST-Core1-170-His and NS5A-His proteins were mixed with different concentrations of the NS5A inhibitor BMS-790052 (left panel) or the cyclophilin inhibitor CsA (right panel). Captured proteins were analyzed by Western blotting using anti-NS5A antibodies.

We first examined whether the core-NS5A interaction truly occurs in a cellular context. Specifically, we conducted co-immunoprecipitation experiments in human hepatoma Huh7 cells, which overexpress core and NS5A (Figure 2A, top panel) and in Huh7 subgenomic JFH1 replicon cells (SGR-JFH1) [59] transfected with core (Figure 2A, bottom panel). N-terminally-3xFlag-tagged core protein was immunoprecipitated with an anti-Flag antibody and resulting immunocomplexes were examined for NS5A content by Western blotting. To exclude nonspecific interactions with the anti-Flag antibody, lysates from cells overexpressing only 3xFlag-Core (Figure 2A, top panel, lane 1) or NS5A (Figure 2A, top panel, lane 2) were incubated with anti-Flag affinity resin. Overexpressed NS5A was efficiently co-immunoprecipitated with core (Figure 2A, top panel, lane 3). Similarly, subgenomic NS5A was efficiently co-immunoprecipitated with core (Figure 2A, bottom panel, lane 1), but not in the absence of core (Figure 2A, bottom panel, lane 2). Although we observed a slight precipitation of NS5A in the absence of core (Figure 2A, bottom panel, lane 2) in some experiments, we considered that small amount as background. Our results confirm that the interaction between core and NS5A occurs in a cellular context (Figure 2A).

To demonstrate that the interaction between core and NS5A is direct, we expressed and purified both proteins from *E.coli* as fusion molecules with a C-terminal hexahistidine tag. In addition, core was N-terminally linked to a GST moiety to allow GST pulldowns. Using that system, we were able to map the core regions required for NS5A binding. Specifically, we generated a series of N- and C-terminally truncated forms of GST-Core-His and used them as bait to pulldown full-length NS5A-His (Figure 2B). GST-Core1-170-His, containing both domains D1 and D2, captured NS5A-His even more efficiently than GST-CypA that we used as positive control since we previously reported that cyclophilin A (CypA) binds directly to NS5A [64]. In contrast, GST alone did not capture NS5A. We observed differences of NS5A-His capture between truncated forms of core. Our results indicated that the core-NS5A interaction was driven by the D1 domain (1–121) of core, but not D2 domain (122–170). The D1 domain of core is composed of three highly charged amino acids clusters (Figure 1A). Pulldowns narrowed down the major core-NS5A interaction region to the second basic cluster (BD2) located between amino acids P38 and K74 of D1 domain of core (Figures 2B and C). Indeed, GST-Core-BD2-His pulled down NS5A-His as efficiently as GST-Core1-170-His, whereas GST-Core75-170-His and GST-Core122-170-His pulled down NS5A-His weakly or even not at all (Figure 2B). RNase, DNase (Figure 2D, left panel) or benzonase nuclease (Firgure 2D, right panel) treatment of recombinant GST-Core1-170-His and NS5A proteins did not prevent their contact, suggesting that the interaction between core and NS5A was not mediated by contaminating nucleic acids. GST-CypA was used as positive control (Figure 2D). Together these results suggest that core and NS5A interact directly and mainly via the BD2 region of core, although we cannot exclude the possibility that BD1 and BD3 also contribute to the binding.

After demonstrating direct contacts between core and NS5A, we tested the inhibitory effect of the NS5A inhibitor BMS-790052 on core-NS5A interactions (Figure 2E). The CypA inhibitor cyclosporine A (CsA) was used as positive control for the pulldown assay since we previously showed that CsA disrupts CypA-NS5A interactions [64]. We found that CsA does not interfere with the capture of full-length NS5A-His by GST-Core1-170-His (Figure 2E, right panel) while efficiently preventing GST-CypA-NS5A contacts. This is in accordance with the fact that cyclophilin inhibitors target the enzymatic pocket of CypA and not NS5A. Interestingly, the highly potent NS5A inhibitor BMS-790052 fails to block core-NS5A interactions even at a concentration of 10 μM, suggesting that the mechanism of its antiviral action does not involve the disruption of contacts between core and NS5A (Figure 2E, left panel).

### Alanine substitutions of basic residues R50, K51, R59 and R62 in core impair NS5A binding

**Figure 3:**
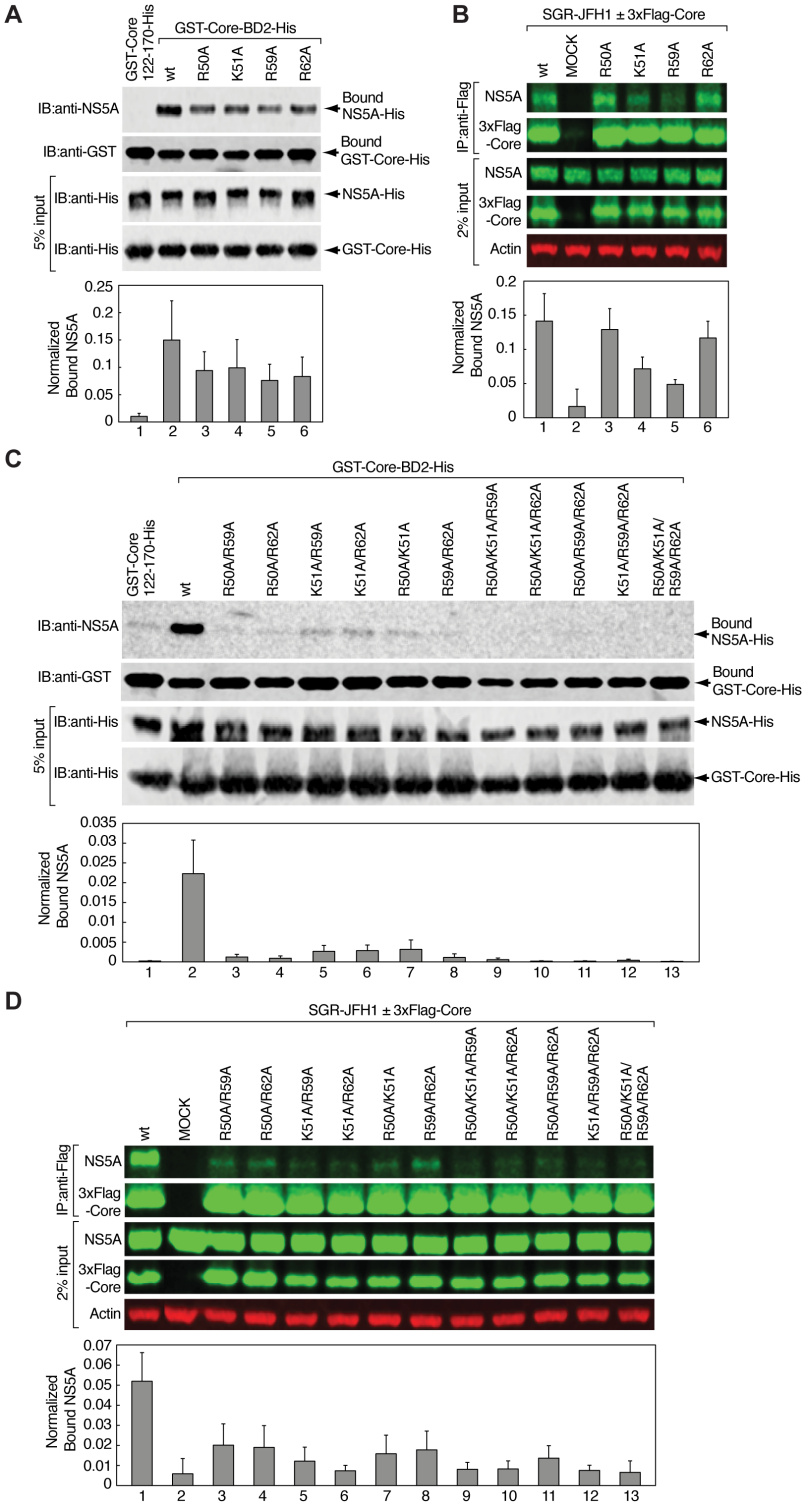
Basic residues R50, K51, R59 and R62 in core are essential for NS5A binding. A) Pulldowns using GST-Core122-170-His (negative control), wild-type GST-Core-BD2-His (wt) or single alanine GST-Core-BD2-His mutants (R50A, K51A, R59A, and R62A) as bait to capture full-length NS5A-His. Bound proteins were analyzed by Western blotting using anti-NS5A and anti-GST antibodies. Input (5%) used for each GST pulldown was probed with anti-His antibodies. The membrane was visualized using respective IRDye secondary antibodies and bound material quantified on the Odyssey IR imaging system. Amount of captured NS5A-His was expressed as IR signal of bound NS5A normalized to the amount of precipitated GST-Core proteins in each lane from three independent experiments. B) Flag co-immunoprecipitations in SGR-JFH1 cells transfected with an expression vector for wild-type 3xFlag-Core (wt), single alanine mutants (R50A, K51A, R59A, and R62A) or empty pcDNA3 plasmid. After immunoprecipitation with anti-Flag antibodies, bound material was eluted with 3xFlag peptide and analyzed by Western blotting with anti-NS5A and anti-Core antibodies. Input of whole-cell lysate (2%) used for each co-immunoprecipitation was probed with anti-NS5A, anti-Core and anti-Actin antibodies. The membrane was visualized using respective IRDye secondary antibodies and bound material quantified on the Odyssey IR imaging system. Amount of co-immunoprecipitated NS5A was expressed as IR signal of bound NS5A normalized to the amount of immunoprecipitated 3xFlag-Core proteins and protein expression levels in each lane from three independent experiments. C) Pulldowns using GST-Core122-170-His (negative control), wild-type GST-Core-BD2-His (wt) or double, triple and quadruple alanine core mutants (listed in Figure 1B) as bait to capture full-length NS5A-His. Bound proteins were analyzed by Western blotting as described above (A). D) Flag co-immunoprecipitations in SGR-JFH1 cells transfected with wild-type 3xFlag-Core (wt), double, triple and quadruple alanine core mutants (listed in Figure 1B) or empty pcDNA3 plasmid. Co-immunoprecipitated proteins were analyzed by Western blotting as described above (B).

Because the four basic residues of core R50, K51, R59, and R62, out of eighteen tested, were previously shown to be crucial for the production of infectious HCV particles [68], and that they were located in the BD2 domain of core, we asked in this study whether these amino acids are also required for NS5A binding. To test this hypothesis, we employed two different strategies (Figure 1B). The first one was based on GST pulldowns using recombinant GST-Core-BD2-His as bait to capture recombinant NS5A-His. The second strategy was based on Flag co-immunoprecipitation experiments using 3xFlag-Core transfected into SGR-JFH1 cells. For both strategies, we generated a series of mutants in GST-BD2-His as well as 3xFlag-Core constructs with single, double, triple and quadruple alanine substitutions R50A, K51A, R59A, and R62A. Amount of bound NS5A in both strategies was analyzed by quantitative, two-color Western blot detection system with infrared (IR) fluorescence that was shown to provide high sensitivity and a wide quantitative linear range with low blot-to-blot variability in signal [69] (Figure 3).

Single alanine substitutions resulted in an average reduction of 37% for R50A, 34% for K51A, 49% for R59A, and 45% for R62A in NS5A binding as revealed by GST pulldowns (Figure 3A) from three independent experiments. GST-Core122-170 was used as negative control. The same single substitutions in a cellular context resulted in much greater variations between basic residue mutants (Figure 3B). K51A and R59A showed an average reduction of 49% and 65% in NS5A binding, respectively, while R50A and R62A showed only 8% and 17% reduction, respectively. The observed discrepancies between the two strategies could simply be explained by complex and more relevant nature of protein co-immunoprecipitation within a cellular context. We then decided to analyze all possible mutation combinations of the four basic amino acids for NS5A binding. Importantly, GST pulldowns demonstrated an average decrease in NS5A binding of 86%–99% for all double, triple, and quadruple GST-BD2-His mutants (Figure 3C). Similar results were obtained using full-length 3xFlag-Core mutants transfected into SGR-JFH1 cells with as much as 61% and 87% decrease in capture of subgenomic NS5A compared to wild-type core (Figure 3D). Note, the background in GST pulldowns (level of bound NS5A by GST-Core122-170, Figures 3A and C) is about 10% lower than in the cellular context (level of bound NS5A to Flag resin only in Mock cells, Figures 3B and D). Together these data indicate that mutating only two basic residues in core sufficed to dramatically abrogate core-NS5A interactions (Figures 3C and D). Our results strongly suggest that the four basic residues in HCV core, that were previously shown to be critical for viral infectivity, are essential for NS5A binding.

### Single, double, triple, and quadruple mutations of core residues R50, K51, R59 and R62 have no effect on viral RNA replication, but abolish the HCV particle release

We next aimed to define the exact stage of the HCV life cycle that could be regulated by core-NS5A interactions mediated by four core basic residues R50, K51, R59, and R62. To address this issue, we constructed a series of single, double, triple, and quadruple substitutions in the context of full-length HCV genome. We used a Luc-JFH1 construct [12], [61] that contains the firefly luciferase gene as reporter to quantitatively measure viral RNA replication. In order to be able to create a stable cell line expressing Luc-JFH1 replicon, which would support replication and production of infectious particles, we introduced a neomycin resistance gene right after the luciferase gene. This construct was named JFH1-Luc/Neo and often referred as wild-type (wt) throughout the manuscript (Figure 1C). Additionally, to be able to conduct Flag-core co-immunoprecipitations in cells expressing genomic JFH1-Luc/Neo, a single Flag tag (DYKDDDDK) with a short linker (SGS) was inserted between the amino acids S2 and T3 of core leading to a new JFH1-Luc/Neo-Core-Flag genomic construct (Figure 1C). The insertion of a small tetracysteine tag of a similar length in this exact position of core was previously reported to be well tolerated for functional viral assembly and infectivity in the HCV Jc1 clone [19].

**Figure 4:**
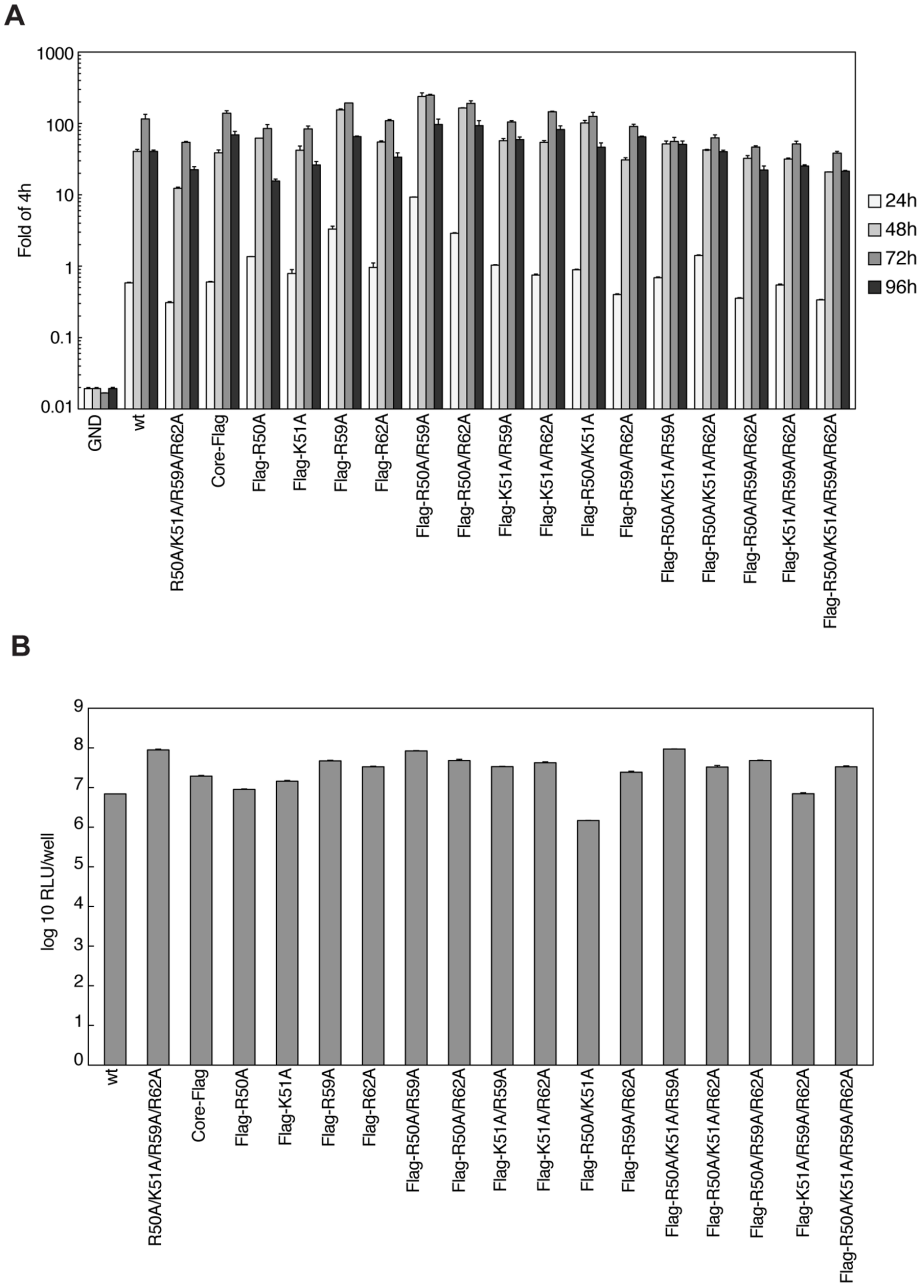
Replication of wild-type and core mutant JFH1-Luc/Neo and JFH1-Luc/Neo-Core-Flag full-length genomes. A) Replication of JFH1-Luc/Neo-GND (negative control with point mutation changing GDD motif in NS5B into GND), JFH1-Luc/Neo (wt), its quadruple core mutant (R50A/K51A/R59A/R62A), JFH1-Luc/Neo-Core-Flag and its given mutants in transfected Huh7.5.1 cells was determined by luciferase activity measurement. Cells from duplicate wells were lysed at given time points post-electroporation. Values are expressed relative to the reporter activity measured at 4 h. Mean values of triplicate measurements and standard errors are presented. B) The replication fitness measured by luciferase activities of stable cell lines created with the same plasmids used above (A). Cells were seeded in 6-well plates in duplicates at the concentration of 10^5^ cells per well, and luciferase activity measured after 72 h. Values are expressed as log_10_ of relative light units (RLU) per single well. Mean values of triplicate measurements and standard errors are presented.

First, we examined the replication of wild-type JFH1-Luc/Neo and its quadruple mutant as well as JFH1-Luc/Neo-Core-Flag and its single, double, triple, and quadruple core mutants. Replication efficiency was measured by luciferase activity at 4, 24, 48, 72, and 96 h post-electroporation. Values were expressed relative to the 4 h luciferase measurements. Our results indicated that all core mutations had no impact on viral RNA replication (Figure 4A). In contrast, a genome containing a lethal mutation in the NS5B RNA-dependent RNA polymerase motif GDD to GND (JFH1-Luc/Neo-GND) did not replicate. We then created stable cell lines expressing all these genomic constructs (except negative control GND) using G418 selection. Importantly, stably expressed viral genomes maintained RNA replication at similar levels (Figure 4B). The main reason why we chose to create stable cell lines expressing genomic JFH1-Luc/Neo was that we observed a poor expression of viral proteins after transient transfections (data not shown). Moreover, the maintenance of the mutations in all of the stable cell lines was verified by RT-PCR and sequencing.

**Figure 5:**
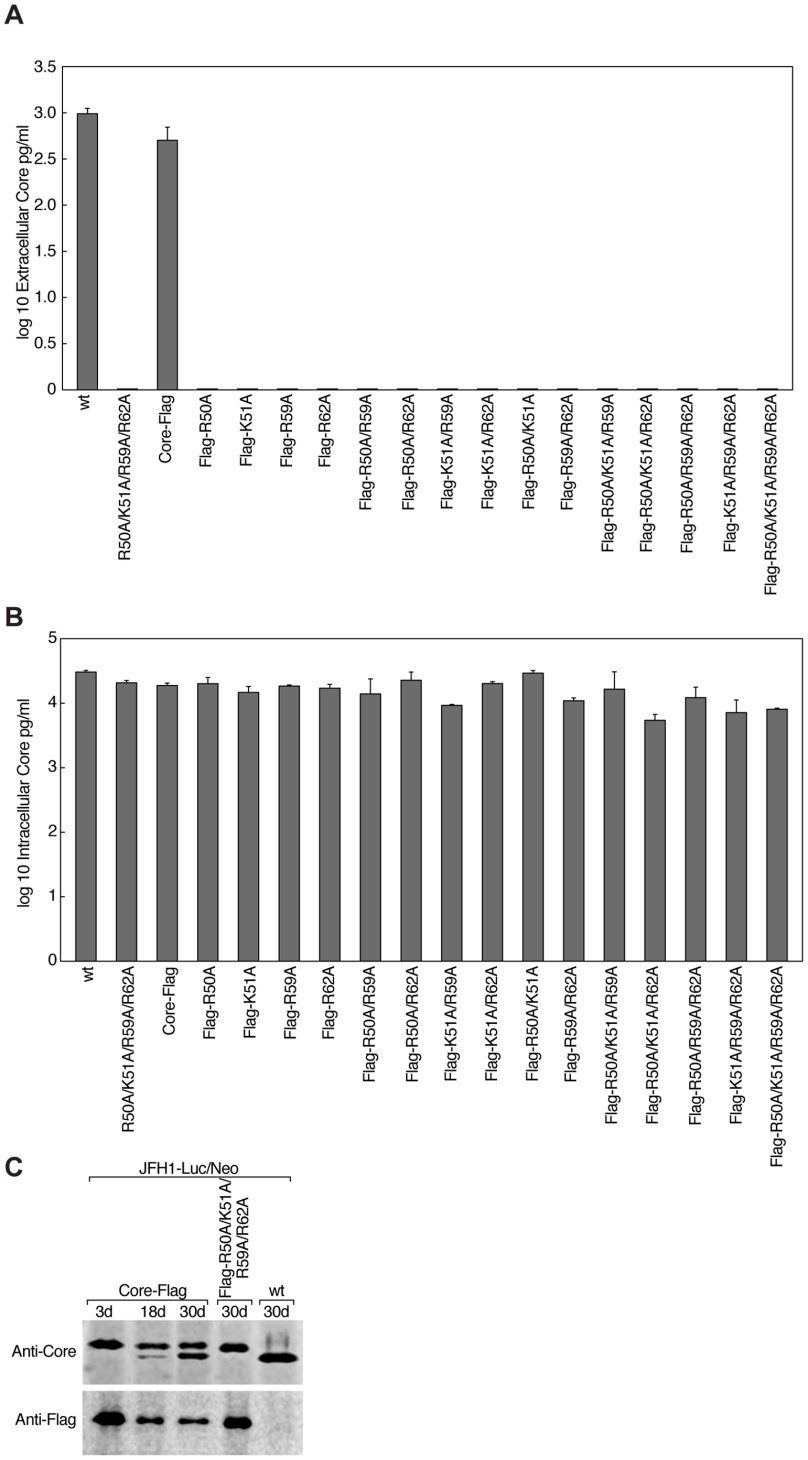
Analysis of extra- and intracellular core in stable cell lines expressing mutant full-length JFH1 genomes. A) Extracellular core levels of stable cell lines expressing JFH1-Luc/Neo (wt), its quadruple core mutant (R50A/K51A/R59A/R62A), JFH1-Luc/Neo-Core-Flag and its given core mutants were quantified by ELISA. Cells were seeded in 6-well plates in triplicates at the concentration of 10^5^ cells per well and grown for 72 h in 3 ml of complete DMEM. Levels of extracellular core were expressed as log_10_ of pg/ml of cell culture medium. Mean values of triplicates and standard errors are presented. B) Intracellular core levels were quantified by ELISA using cells plated in the experiment described above (A). Cells were lysed with 0.3 ml of Cell Culture Lysis Reagent. Intracellular core levels were expressed as log_10_ of pg/ml of cell lysate. Mean values of triplicates and standard errors are presented. C) A long-term stability analysis of core in stable cell lines expressing JFH1-Luc/Neo-Core-Flag, its quadruple core mutant (Flag-R50A/K51A/R59A/R62A) as well as JFH1-Luc/Neo (wt). Cells were kept under neomycin selection for a month and passaged every 3 days. Cell lysates from the first passage (3 d, three days), the sixth passage (18 d, eighteen days) and the tenth passage (30 d, thirty days) were analyzed by Western blotting with anti-Core and anti-Flag antibodies.

To analyze viral particle production by cells stably expressing wild-type and mutant HCV genomes, we quantified extra- and intracellular core levels by ELISA (Figure 5). In contrast to JFH1-Luc/Neo and JFH1-Luc/Neo-Core-Flag, all stable cell lines expressing HCV genomes with single, double, triple, and quadruple core mutants did not produce detectable amounts of core in the cell culture supernatant, indicating that the release of HCV particles was abrogated (Figure 5A). Comparable amounts of intracellular core were detected in all stable cell lines, indicating that the RNA replication and the translation were not affected by alanine substitutions of core residues R50, K51, R59, and R62 (Figure 5B). This is in accordance with our previous observation that wild-type and core mutant viruses exhibited similar intracellular levels of luciferase activities (Figure 4B). The fact that the mutant core proteins were recognized by ELISA, suggests that the alanine substitutions did not influence the stability and overall conformation of core. In addition, we confirmed the long-term (30 days) core protein stability in G418-resistant JFH1-Luc/Neo-Core-Flag, JFH1-Luc/Neo-Core-Flag-R50A/K51A/R59A/R62A and JFH1-Luc/Neo cell lines by Western blotting (Figure 5C). After six passages (18 days), we noticed the presence of a second lower molecular band of core in JFH1-Luc/Neo-Core-Flag expressing cells that was not recognized by the anti-Flag antibody, we also noticed that its intensity increased after ten passages (30 days). We therefore did not maintain our stable cell lines in culture longer than 2 weeks for all experiments.

### Mutants with core substitutions R50A, K51A, R59A, and R62A do not produce infectious particles

**Figure 6:**
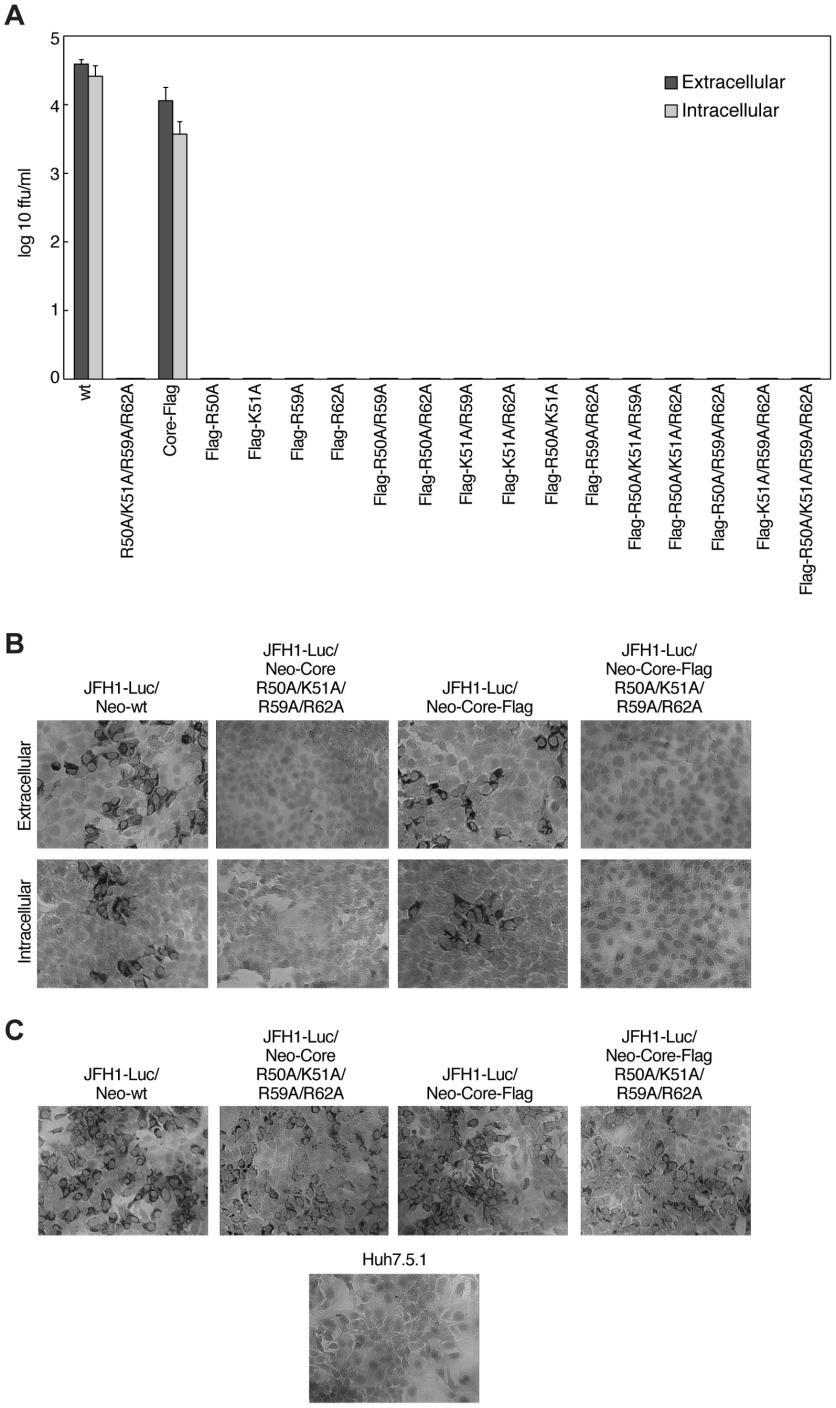
Mutants with core substitutions R50A, K51A, R59A and R62A do not produce infectious HCV particles. A) Extra- and intracellular infectivity of stable cell lines expressing JFH1-Luc/Neo-wt, its quadruple core mutant (R50A/K51A/R59A/R62A), JFH1-Luc/Neo-Core-Flag and its given mutants was determined by a focus-forming assay. Levels of extra- and intracellular infectivity were expressed as log_10_ of focus-forming units (ffu) per ml of supernatant or cell lysate, respectively. Mean values of triplicates and standard errors are presented. B) Representative light microscopic pictures of infectious foci in naïve Huh7.5.1 cells exposed to extra- and intracellular HCVcc particles from the experiment described above (A). Cells were counter stained with hematoxylin to visualize the nuclei. The magnification is 20x. C) Stable cell lines expressing JFH1-Luc/Neo-wt, its quadruple core mutant (R50A/K51A/R59A/R62A), JFH1-Luc/Neo-Core-Flag and its quadruple core mutant (Flag-R50A/K51A/R59A/R62A) were seeded in 96-well plate and the standard immunostaining procedure for a focus-forming assay was performed directly on them. Cells were counter stained with hematoxylin to visualize the nuclei. The magnification is 20×.

A previous study showed that the formation of viral particles occurs within the cell and that production of intracellular infectious viral particles precedes secretion of infectious viruses. Secreted particles differ in buoyant density from intracellular particles, suggesting that they go through a maturation process that alters particle density [66]. We thus sought to investigate the effect of core mutations within JFH1-Luc/Neo or JFH1-Luc/Neo-Core-Flag on the production of infectious intracellular particles. We measured both extra- and intracellular virus titers over a 72 h time-course by a focus-forming assay for all stable cell lines expressing JFH1-Luc/Neo and JFH1-Luc/Neo-Core-Flag as well as their given core mutants (Figures 6A and B). No infectious extracellular particles were detected for all of the mutants. This was expected since they failed to be released (Figure 5A). Interestingly, the intracellular particles of core mutants were not infectious in contrast to wild-type intracellular particles (Figures 6A and B). We then confirmed that the antibody used for the focus-forming assay – the anti-Core monoclonal antibody C7-50 - recognizes all mutant core proteins (Figure 6C). Taken together, our data indicated that mutating R50, K51, R59, and R62 in core abolished the infectivity and/or assembly of intracellular particles.

### Mutating all four residues R50, K51, R59 and R62 does not alter the association of core and NS5A with LDs, but impairs core-NS5A interactions

**Figure 7:**
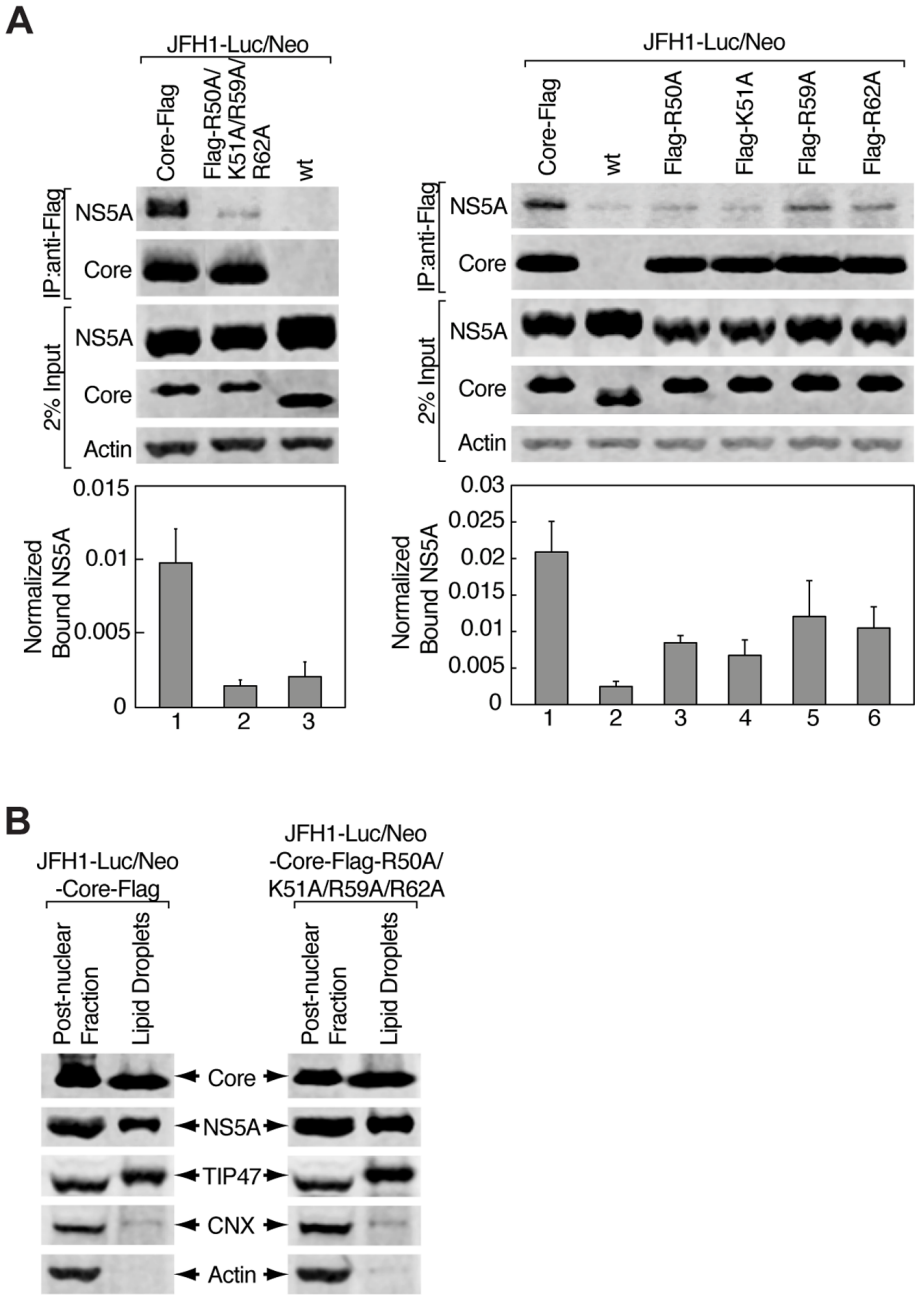
Core substitutions R50A, K51A, R59A and R62A in JFH1-Luc/Neo-Core-Flag full-length genome impair core-NS5A interaction, but do not alter their associations with LDs. A) Flag co-immunoprecipitations in stable cell lines expressing JFH1-Luc/Neo-Core-Flag, its quadruple core mutant Flag-R50A/K51A/R59A/R62A and JFH1-Luc/Neo-wt (left panel) or single alanine mutants Flag-R50A, Flag-K51A, Flag-R59A, and Flag-R62A (right panel). After immunoprecipitation with anti-Flag antibodies, bound material was eluted with 3xFlag peptide and analyzed by Western blotting with anti-NS5A and anti-Core antibodies. Input of whole-cell lysate (2%) used for each co-immunoprecipitation was probed with anti-NS5A, anti-Core and anti-Actin antibodies. Amount of co-immunoprecipitated NS5A was expressed as IR signal of bound NS5A normalized to the amount of immunoprecipitated 3xFlag-Core proteins and protein expression levels in each lane from three independent experiments. B) LD fraction from stable cell lines expressing JFH1-Luc/Neo-Core-Flag or its quadruple core mutant (Flag-R50A/K51A/R59A/R62A) was isolated by a differential membrane flotation method. Proteins from post-nuclear fraction (input) and proteins associated with LD fraction were analyzed by Western blotting with antibodies specific to Core, NS5A, TIP47, CNX (calnexin) and Actin.

To prove that core indeed interacts with NS5A through the four basic residues R50, K51, R59, and R62 in the context of the infectious HCVcc system, we performed Flag co-immunoprecipitation experiments in cells stably expressing wild-type and quadruple as well as single core mutant full-length HCV genomes. As demonstrated in Figure 7A, NS5A from JFH1-Luc/Neo-Core-Flag expressing cells was co-immunoprecipitated with wild-type core (Figure 7A, left and right panel, lane 1). Core-Flag-R50A/K51A/R59A/R62A showed an average reduction of 85% in NS5A binding (Figure 7A, left panel), while single core mutants showed a decrease of 60% (Flag-R50A), 68% (Flag-K51A), 42% (Flag-R59A), and 50% (Flag-R62A) in capture of NS5A comparing to wild-type core (Figure 7A, right panel). For additional specificity of the core-NS5A interaction, we used a stable cell line expressing JFH1-Luc/Neo and showed that wild-type core deprived of its Flag tag as well as NS5A were not immunoprecipitated (Figure 7A; left panel, lane 3; righ panel, lane 2). The results confirmed that mutations in positions 50, 51, 59, and 62 of core impaired its ability to interact with NS5A in a full genomic HCV system.

To test whether core-NS5A interactions, mediated by the four basic amino acids of core, regulate the association of both proteins with LDs, we isolated the LD fraction with a differential membrane flotation method (Figure 7B). TIP47 - a marker of LDs [52], [67] - was enriched in the LD fraction, whereas the ER marker calnexin and the cytosolic marker actin were absent, confirming the purity of the LD fraction. We found that core as well as NS5A from both wild-type and quadruple mutant HCV were present in the LD fraction, demonstrating that the mutations of the four basic core residues did not alter the subcellular localization of the two viral proteins and suggesting that the core-NS5A interaction is not a requirement for the association of core and NS5A with LDs.

### Sucrose density gradient ultracentrifugation analysis of cells expressing wild-type and quadruple mutant viruses reveals similar viral and host protein profiles

Current biochemical and morphological characterization of infectious HCVcc particles is mostly based on studies using affinity purification of secreted virus particles combined with electron microscopy analysis [56] or electron cryomicroscopy of particles purified by density gradient ultracentrifugation [70]. Both approaches revealed that infectious HCV particles are heterogenous in terms of their structures and buoyant densities. It has been suggested that the higher-density particles are non-enveloped RNA-containing capsids and are unlikely to be infectious [70].

**Figure 8:**
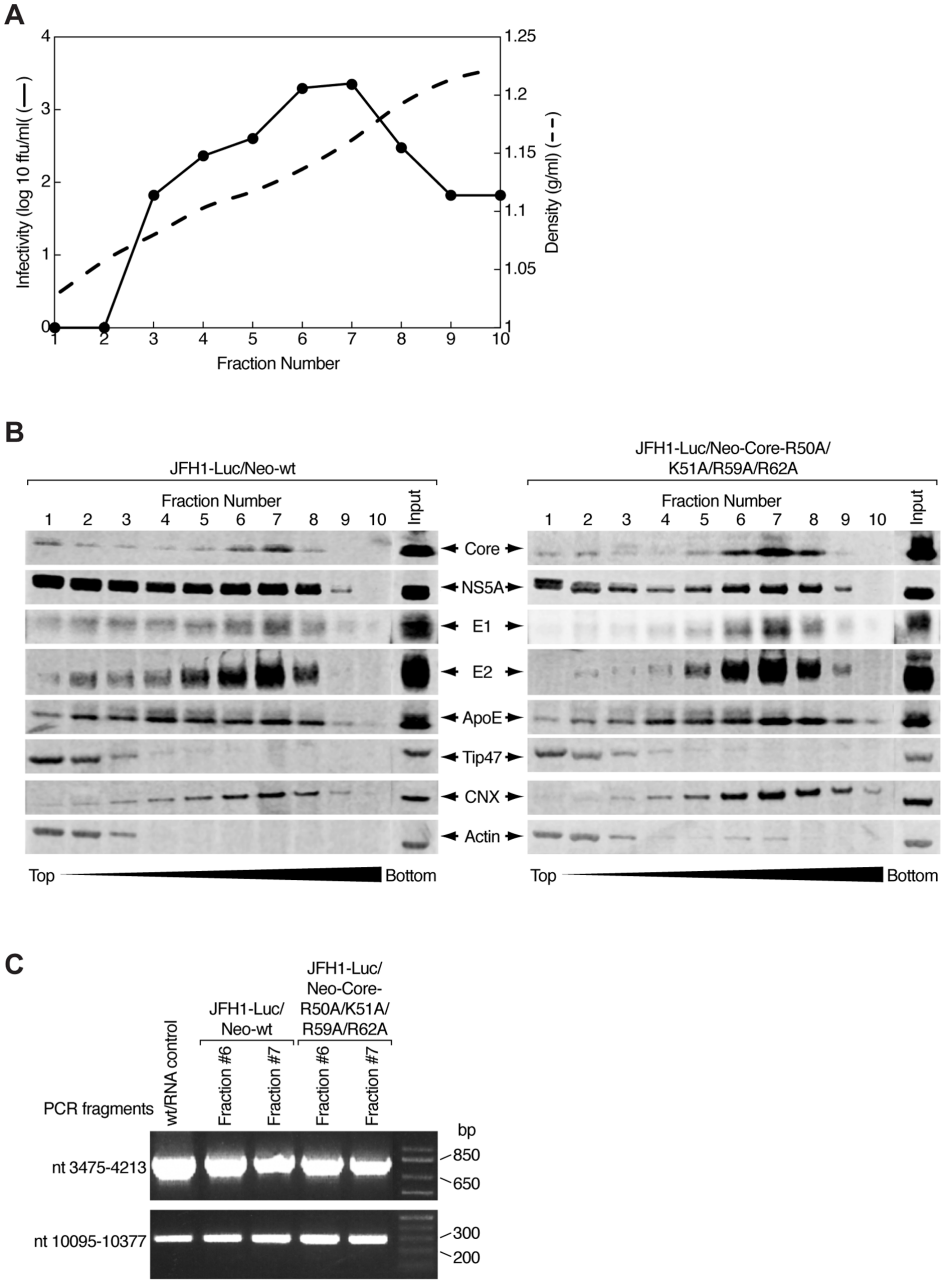
Analysis of fractionated cells expressing wild-type or non-infectious quadruple mutant viruses. A) Representative buoyant density profile of viral infectivity in cell lysate of stable cell line expressing JFH1-Luc/Neo-wt in continuous 10% to 60% sucrose gradient. Infectivity (black circles) was determined by a focus-forming assay on Huh7.5.1 cells and expressed as log_10_ of focus-forming units (ffu) per ml of each fraction. Fraction densities were determined by measuring the sucrose content in similar fractions of a control gradient with a refractometer. The dotted line represents the density (g/ml) measured in each fraction. B) Western blotting analysis following sucrose gradient ultracentrifugation of cell lysates from stable cell lines expressing JFH1-Luc/Neo-wt and its quadruple core mutant (R50A/K51A/R59A/R62A). Cytoplasmic lysates were deposited onto the top of a continuous 10% to 60% sucrose gradient and centrifuged at 29,000 rpm for 16 h at 4°C. Samples of 1 ml were collected from the top of the gradient and 10 µl of each fraction was analyzed by Western blotting with antibodies specific to Core, NS5A, envelope glycoproteins E1 and E2, ApoE (apolipoprotein E), TIP47 (tail interacting protein of 47 kDa), CNX (calnexin) and Actin. C) RT-PCR analysis of fractions 6 and 7 from 10%-60% sucrose gradients described above (B). RNA was extracted via acidic phenol-chloroform extraction of the sucrose fractions (200 µl) followed by LiCl precipitation. RNA (300 ng) from each fraction was tested with two pairs of oligonucleotides: one spanning EMCV-IRES and core nucleotide sequence (positions 3475 and 4213 of JFH1-Luc/Neo replicon) and the other spanning NS5A nucleotide sequence (positions 10092 and 10377 of JFH1-Luc/Neo replicon).

To further elucidate the non-infectious nature of intracellular quadruple mutant particles, we performed sucrose density gradient ultracentrifugation analysis of cell lysates from both JFH1-Luc/Neo and JFH1-Luc/Neo-Core-R50A/K51A/R59A/R62A in order to compare their protein profiles (Figure 8). For cells expressing wild-type virus, we obtained a single infectivity peak in fractions 6 and 7 (Figure 8A), corresponding to a density range of 1.14-1.16 g/ml that was in line with previously published data [66]. As expected, we did not detect any residual infectivity associated with fractions from cells expressing the quadruple core mutant virus (data not shown). Western blot analysis of collected sucrose fractions after ultracentrifugation showed similar migration and content profiles of viral and cellular proteins between the two stable cell lines (Figure 8B). Specifically, we observed that structural viral components (core, E1, and E2) co-migrated mostly in fractions 6 and 7, where the viral RNA from both stable cell lines was detected (Figure 8C). The most intense bands of calnexin, the ER marker, were also displayed in fractions 6 and 7, suggesting that the majority of viral assembly sites were in close proximity to cellular membranes, most probably to subsequently allow particles to go through the secretory pathway. TIP47, the LD marker, was mostly localized in lower density fractions at the top of sucrose gradient. NS5A and ApoE distributions were more dispersed when comparing them with those of other viral proteins, but still overlapping with fractions 6 and 7, corresponding to the peak of infectivity. Thus, ultracentrifugation analysis of intracellular HCV particles failed to demonstrate differences in profiles of co-fractionated proteins between the non-infectious mutant and the infectious wild-type virus.

### Domain I of NS5A rather than domain III contains the core-binding site

**Figure 9:**
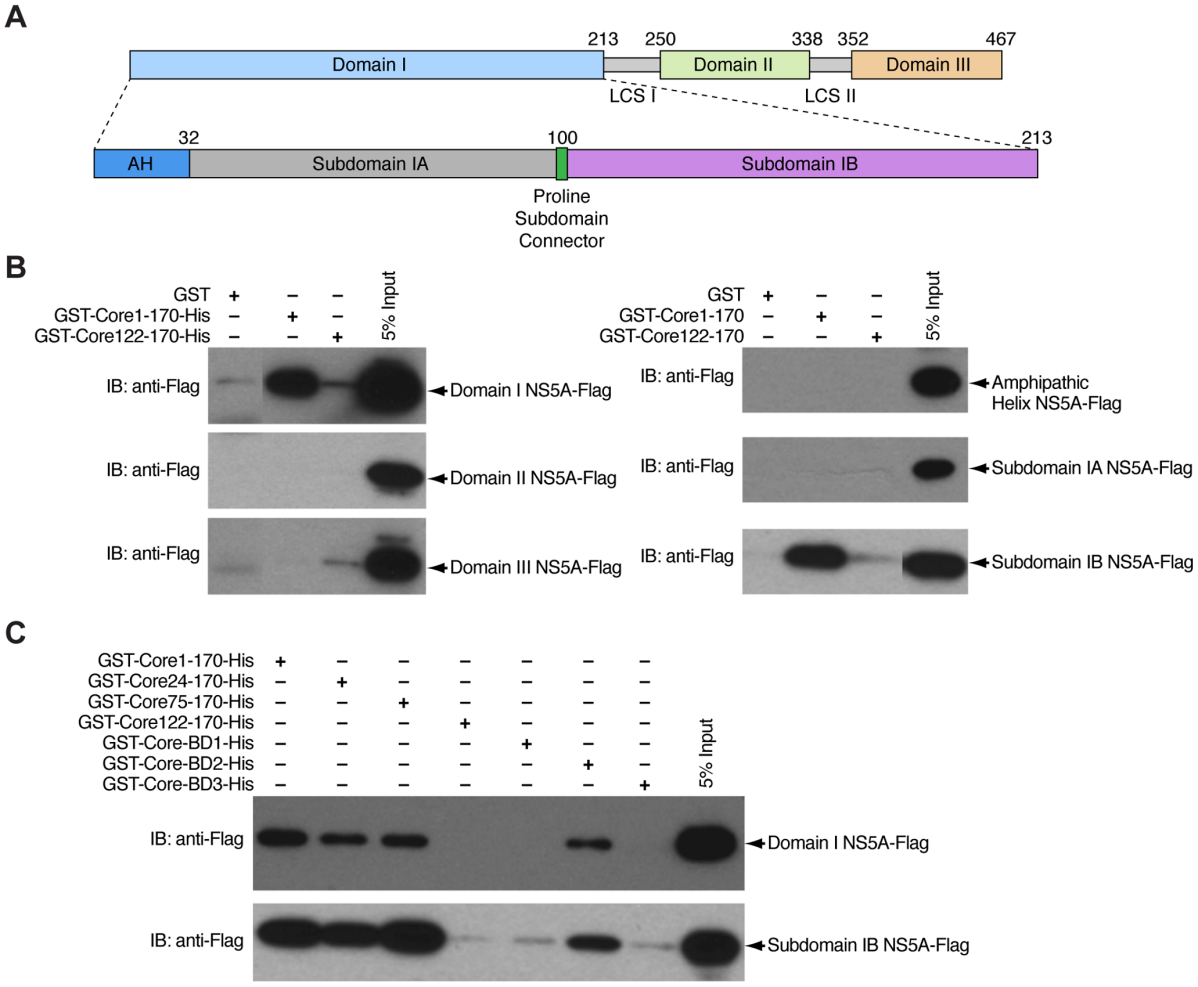
Domain I of NS5A binds to core. A) NS5A is composed of three domains (Domain I, II, and III) separated by low-complexity sequences (LCSI and LCSII). Domain I of NS5A is composed of a 32-amino-acid N-terminal amphipathic helix (AH), subdomain IA (33–100) and subdomain IB (101–213). B) Mapping of NS5A regions required for binding to core. GST and GST-Core122-170-His (negative controls) or GST-Core1-170-His were used as bait to pulldown domain I NS5A-Flag, domain II NS5A-Flag and domain III NS5A-Flag (left panel) or amphipathic helix NS5A-Flag, subdomain IA NS5A-Flag, and subdomain IB NS5A-Flag (right panel). Captured proteins and 5% of input were analyzed by Western blotting using anti-Flag antibodies. C) GST and truncation forms of GST-Core-His were used as bait to pulldown domain I NS5A-Flag and subdomain IB NS5A-Flag. Captured proteins and 5% of input were analyzed by Western blotting using anti-Flag antibodies.

NS5A can be separated into three domains: domain I (1–213 aa), domain II (250–338 aa), and domain III (352–467 aa) (Figure 9A). Domains I and II are playing key roles in replication of viral RNA, whereas domains I and III are playing key roles in viral assembly. Mutations in domain I of NS5A that abolished LDs localization (APK99–101AAA and PPT102–104AAA) blocked viral assembly and infectious particle production, likely due to the inability of HCV replication complexes to be recruited to the sites of assembly [32]. Removal of most of the domain III of NS5A did not impair its targeting to LDs, but led to impaired virion assembly and the truncated NS5A protein was found on LDs that do not contain core [41]. Mutations of serine residues in domain III of NS5A at positions 2428, 2430, and 2433 (amino acid positions within the HCV polyprotein corresponding to the positions 452, 454 and 457 of full-length NS5A) reduced its basal phosphorylation, caused NS5A mislocalization, impaired virion assembly and disrupted core-NS5A interactions [34]. Since we were not able to demonstrate by our pulldown assays that alanine substitutions of three serine residues of NS5A at positions 2428, 2430, and 2433 impaired core-NS5A interaction (data not shown), we decided to re-investigate the core-binding site within NS5A.

To examine which regions of NS5A are responsible for direct interactions with core, we expressed and purified domain I NS5A-Flag, domain II NS5A-Flag and domain III NS5A-Flag and performed GST pulldowns using them as prey (Figure 9B, left panel). GST-Core-His proteins were used as bait. GST-Core1-170-His captured efficiently domain I, but not domain II and III of NS5A. GST and GST-Core122-170-His, used as negative controls, did not pulldown any of the NS5A proteins. We next generated and purified fragments of NS5A domain I that can be further separated into N-terminal amphiphatic helix (1–32 aa), subdomain IA (33–100 aa), and subdomain IB (101–213 aa) (Figure 9A) [71]. GST-Core1-170-His pulled down subdomain IB NS5A-Flag, but not amphipathic helix NS5A-Flag or subdomain IA NS5A-Flag, whereas negative controls (GST and GST-Core122-170-His) did not pulldown any proteins (Figure 9B right panel). Additionally, we conducted GST pulldowns using different truncation constructs of GST-Core-His as bait and domain I or subdomain IB NS5A-Flag as pray (Figure 9C). The results confirmed that domain I of NS5A and its subdomain IB interact with the D1 (1–121) domain of core, but not the D2 domain (122–170) of core. Interestingly, both domain I and subdomain IB of NS5A bound to the BD2 (38-74) of core, but not to BD1 (2–23) or BD3 (101–121). These data suggest that domain I of NS5A rather than domain III is responsible for direct core-NS5A interactions.

## Discussion

The HCV assembly process involves interactions between viral structural and nonstructural proteins and coordinated actions of cellular proteins [72]. Systemic approaches determined that for the overall cell interactome, core, NS3 and NS5A were the most connected viral proteins with 76, 214, and 96 host protein partners, respectively [73]. Among nonstructural proteins, NS5A seems to be the most characterized in terms of its role in viral assembly. Mutagenesis studies found that the deletions of domain III of NS5A disrupted the production of infectious virus particles without altering the efficiency of HCV RNA replication [40], [41]. Interestingly, the deletions of domain III of NS5A did not impair NS5A targeting into the surface of LDs, but disrupted its co-localization with core on the same LDs [41]. However, a direct interaction between core and NS5A in infected cells was not observed [41]. Moreover it has been demonstrated that a single substitution S457A in NS5A (S2433A, amino acid positions within the HCV polyprotein) abrogated infectious virion assembly and suggested that phosphorylation at this position regulates the production of infectious virus [40]. By contrast, the alanine scanning mutagenesis revealed, that within the same region of NS5A (C-terminal region of domain III), substitutions of at least two serine residues at positions 2428, 2430, and 2433 (amino acid positions within the HCV polyprotein) were required to impair both virion production and core-NS5A interactions [34]. In the same study, alanine substitutions of all three serine residues abolished NS5A and core co-localization on LDs as well as the association of viral RNA with core [34]. While there is no clear explanation for the differences observed in these three studies of NS5A, it may be important that Appel et al. [41] studied the assembly of the Jc1 virus, Tellinghuisen et al. [40] studied J6/JFH1 chimera and Masaki et al. [34] used the wild-type JFH1 strain. Another study confirmed that the triple serine mutant of NS5A decreased HCV infectivity, but linked this effect to the stabilization function of phosphorylated forms of NS5A on NS2 dotted structures that favors the assembly process [50]. An independent study also observed lack of NS5A colocalization with core in cells infected with a double serine NS5A mutant virus (S2428/2430A) [39].

Importantly, prior to this study, there were no data in the literature, showing which amino acids of core are required for its contact with NS5A and their importance in HCV particle infectivity and assembly. Comprehensive mutagenesis studies of the core region containing amino acids 57-191 revealed numerous residues critical for infectivity, but not for viral RNA replication [74]. Another core mutagenesis study focused on the two first basic clusters of the D1 domain [68]. The single alanine substitutions of four residues (R50, K51, R59, and R62) within the second cluster were shown to completely abolish viral infectivity. Although the exact stage at which these mutations impacted virus assembly was not identified, defects in core protein stability, colocalization with LDs and NS5A, RNA encapsidation, oligomerization, and envelopment by intracellular membranes were excluded experimentally.

Here we mapped the region of core responsible for NS5A binding. First, we examined that core does interact with NS5A in a cellular context. Our pulldown analyses identified the D1 domain of core as the NS5A-binding site, in which the basic P38-K74 cluster (BD2) appeared to be the major determinant for the interaction. Since it was previously suggested that core-NS5A interactions play an important role in the formation of infectious HCV particles [34], we next proposed that the four N-terminal amino acids of core at positions 50, 51, 59, and 62 located in the BD2 region might be involved in NS5A binding. Using various strategies, we demonstrated that these four basic amino acids were required for NS5A contact. We clearly showed that mutations of only two basic residues sufficed to almost completely block NS5A binding to core. On the other hand, our pulldown analyses showed that BD1 and BD3 are also able to bind full-length NS5A. It seems important to mention here, that BD2 of core protein from genotype 2a strain JFH1 contains fourteen basic amino acids, whereas BD1 – eight, and BD3 – six. Showing that mutations of four basic amino acids in BD2, out of fourteen, completely block the interaction between full-length core and NS5A suggests that the specificity and most probably the nascent protein folding due to the residues R50, K51, R59, and R62 rather than the total number of basic residues play a major role in the NS5A binding. However, in the absence of structural data, we cannot exclude the possibility that mutations of other, also non-basic, residues in the same region will have an impact on core-NS5A interactions. Especially, if we take into consideration how important is for virus infectivity to maintain a close contact between two distant amino acids in HCV core as it has been demonstrated for residues G33 and F24 [75]. Next, we confirmed that core interacts with NS5A through the four N-terminal basic amino acids in cells expressing full-length HCV genome. To understand the mechanism by which core-NS5A interactions can regulate the production of infectious HCV particles, we generated a series of viral constructs with single, double, triple and quadruple substitutions of R50A, K51A, R59A, and R62A in the context of full-length HCV genome. The introduction of mutations in core that impair core-NS5A interactions not only blocked HCV particle release, but also rendered intracellular particles non-infectious. These data suggest that core-NS5A interactions play a vital role in the HCV life cycle.

The infectivity phenotype of core mutants appeared to be much stronger and more definite compared to that of serine NS5A mutants at positions 2428, 2430, and 2433. The reduction in virus release caused by single serine NS5A mutants was approximately 90% 48 h post-transfection, but virus release reached similar levels to those of wild-type 96 h post-transfection [34]. Single alanine substitutions of core amino acids at positions 50, 51, 59, 62 completely disrupted the production of extra- and intracellular infectious virus particles 72 h post-electroporation [68]. We obtained similar results in our stable JFH1-Luc/Neo-Core-Flag cell lines expressing core mutants in which extracellular and intracellular infectivity was abolished. Another study showed that the double S2428/2430A NS5A mutant exhibited a reduction of between 10- to 100-fold in extra- and intracellular virus titers 48 and 72 h post-tranfection, suggesting that the mutant was still able to produce some infectious particles [39]. Our single, double, triple and quadruple core mutants did not produce detectable amounts of extracellular core, indicating a complete block of HCV particle release. Moreover, all of the full-genomic core mutants exhibited a reduction of about 10,000-fold in extra- and intracellular infectivity, with no single focus-forming particle noticed. Since we were not able to demonstrate by our pulldown assays that alanine substitutions of three serine residues of NS5A at positions 2428, 2430, and 2433 impaired core-NS5A interaction (data not shown), we decided to re-examine the core-binding regions in full-length NS5A. Although we cannot exclude that the observed discrepancy is due to differences in the experimental set up, we showed that domain I of NS5A rather than domain III is responsible for direct core-NS5A contacts. Our observations have encouraged us to assume that the core-NS5A interaction investigated in this study, besides different amino acids requirements, has important functions in the assembly of infectious particles.

The assembly events in which core recruits NS5A to LDs and then other NS proteins and viral RNA have been previously proposed [32]. One of the very first model suggested that a newly synthesized HCV RNA bound to NS5A is captured by core via a direct interaction between the two proteins at LDs [34]. Lately, a growing body of evidence implied that other host proteins such as DGAT1 and TIP47 are involved in targeting NS5A carrying viral RNA to the HCV assembly sites on the surface of LDs. Recent publications described an important role of DGAT1 in HCV infection, suggesting that this host protein facilitates the binding of NS5A to core and guides both proteins onto the surface of LDs [53], [54]. The direct interaction between TIP47 and NS5A was also revealed, and importantly, the silencing of TIP47 in cells was shown to be crucial for HCV replication [52]. A very recent study has presented a model where TIP47 through its interaction with NS5A plays a new role in HCV infectivity, possibly by integrating LD membranes into the membranous web [67]. In this model, a close interface between LD and ER of membranous web has been established in the absence of core and other structural proteins. Since NS3, NS5B, and NS5A together with TIP47 and viral RNA were able to access LD membranes without core, it has been suggested that LD membranes are critical parts of the membranous web, independent from viral assembly [67]. Thus, it has become more evident that the viral RNA recruitment from replication complexes to the assembly sites on LDs is a complex process that requires more protein-protein interactions, leading to membranes rearrangements first and subsequently enabling core-NS5A interactions, most probably to transfer viral RNA and initiate its encapsidation. This new model supports our findings and raises the question about the exact role of core-NS5A interactions in HCV infectivity and assembly.

To address this question, we examined the subcellular localization of core and NS5A in cells producing full-length viruses. First, we found that both core and NS5A of wild-type and quadruple core mutant viruses were associated with the LDs fraction, indicating that the four substitutions R50A, K51A, R59A, and R62A in core did not alter their targeting to LDs. Second, we compared the profiles of co-fractionated proteins between wild-type and quadruple core mutant viruses. We did not observe striking differences between them besides the lack of infectivity for the quadruple mutant. The ultracentrifugation analysis was most probably not sensitive and/or adequate enough to point to which step of the assembly process was dysfunctional. At that moment, we can only speculate that an early step of nucleocapsid formation was not affected, based on our fractionation analysis as well as previous studies with single basic core residue mutants showing that all of them were still able to encapsidate viral RNA [68].

Although our data suggest that NS5A is involved in the HCV assembly process through the interaction with core via basic amino acids located in the second basic cluster of the D1 domain, one cannot exclude the possibility that alanine substitutions of R50, K51, R59, and R62 influence the interaction with other proteins or viral RNA. It was demonstrated that the N-terminal part of core (82 first amino acids) was sufficient to trigger the formation of nucleocapsid-like particles *in vitro* when structured RNA was added to the purified protein [76]. In the same study, mutational analysis of a truncated core (C1-82) demonstrated that it is the global positive charge rather than any specific basic residue that is important for the assembly process. However, most of the substitution mutants including positions 50–51, 55–59, and 61–62, were as capable of *in vitro* assembly as was the C1-82 core fragment, thus excluding the possibility that our quadruple mutant core was unable to form nucleocapsid-like particles. Circular dichroism and NMR analysis indicated that core1–82 lacks secondary structure and is highly disordered [77]. As other intrinsically unstructured proteins, it is expected that the D1 domain of core can adopt different conformations depending on the presence of specific cellular partners. This may explain why many viral and host interactions have been mapped to residues within the D1 domain of core. A study of core binding to E1 has suggested that this interaction depends on core amino acids 72 to 91 [26]. It was also proposed that intrinsic disorder feature is important for RNA chaperoning functions of core, although the physiological relevance of this interaction is still not clear [78]. It is important to mention that the arginine and lysine residues between amino acids 39–62 are invariant among all 52 analyzed HCV isolates of different genotypes, suggesting that this region may exert an important function [79]. Despite recent advancements, many aspects of the HCV morphogenesis are still unclear, such as the exact mechanism of the viral genome packaging within the capsid, or the structure and the exact biochemical composition of infectious viral particles. Here, we present that the viral core protein interacts with the nonstructural protein NS5A via four N-terminal basic amino acids that are critical for viral infectivity. Uncovering the molecular details of the core-NS5A interaction mediated by the basic residues R50, K51, R59, and R62 should help the design or the identification of compounds, which efficiently block the formation of infectious HCV particles and may become part of a new therapeutic regimen.
